# Process Intensification of the Continuous Synthesis of Bio-Derived Monomers for Sustainable Coatings Using a Taylor Vortex Flow Reactor

**DOI:** 10.1021/acs.oprd.3c00462

**Published:** 2024-05-09

**Authors:** Matthew D. Edwards, Matthew T. Pratley, Charles M. Gordon, Rodolfo I. Teixeira, Hamza Ali, Irfhan Mahmood, Reece Lester, Ashley Love, Johannes G. H. Hermens, Thomas Freese, Ben L. Feringa, Martyn Poliakoff, Michael W. George

**Affiliations:** † School of Chemistry, University of Nottingham, University Park, Nottingham NG7 2RD, U.K.; ‡ Scale-up Systems Ltd., 23 Shelbourne Road, Dublin 4, D04 PY68, Ireland; § Advanced Research Centre CBBC, Stratingh Institute for Chemistry, Faculty of Science and Engineering, University of Groningen, Nijenborgh 4, 9747 AG Groningen, The Netherlands

**Keywords:** flow chemistry, photo-oxidation, biomass valorization, sustainability, coatings

## Abstract

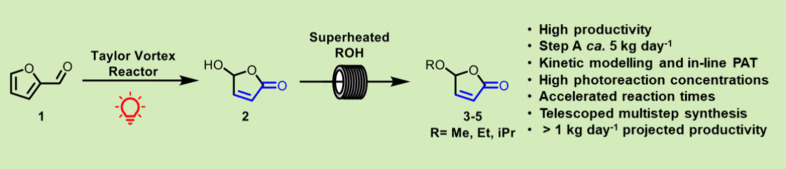

We describe the optimization and scale-up of two consecutive reaction steps in the synthesis of bio-derived alkoxybutenolide monomers that have been reported as potential replacements for acrylate-based coatings (Sci. Adv.2020, 6, eabe002633328241 ). These monomers are synthesized by (i) oxidation of furfural with photogenerated singlet oxygen followed by (ii) thermal condensation of the desired 5-hydroxyfuranone intermediate product with an alcohol, a step which until now has involved a lengthy batch reaction. The two steps have been successfully telescoped into a single kilogram-scale process without any need to isolate the 5-hydroxyfuranone between the steps. Our process development involved FTIR reaction monitoring, FTIR data analysis *via* 2D visualization, and two different photoreactors: (i) a semicontinuous photoreactor based on a modified rotary evaporator, where FTIR and 2D correlation spectroscopy (2D-COS) revealed the loss of the methyl formate coproduct, and (ii) our fully continuous Taylor Vortex photoreactor, which enhanced the mass transfer and permitted the use of near-stoichiometric equivalents of O_2_. The use of in-line FTIR monitoring and modeling greatly accelerated process optimization in the Vortex reactor. This led to scale-up of the photo-oxidation in 85% yield with a projected productivity of 1.3 kg day^–1^ and a space-time yield of 0.06 mol day^–1^ mL^–1^. Higher productivities could be achieved while sacrificing yield (*e.g.*, 4 kg day^–1^ at 40% yield). The use of superheated methanol at 200 °C in a pressurized thermal flow reactor accelerated the second step, the thermal condensation of 5-hydroxyfuranone, from a 20 h batch reflux reaction (0.5 L, 85 g) to a space time of <1 min in a reactor only 3 mL in volume operating with projected productivities of >700 g day^–1^. Proof of concept for telescoping the two steps was established with an overall two-step yield of 67%, producing a process with a projected productivity of 1.1 kg day^–1^ for the methoxybutenolide monomer without any purification of the 5-hydroxyfuranone intermediate.

## Introduction

Our reliance on polymers in daily life is currently dependent on the availability of affordable fossil fuel feedstocks.^1^ There is a growing movement to develop polymers and materials that are sourced from renewable sources to drive toward Net Zero production and a circular economy.^2^ Monomers derived from glycerol,^3^ terpenes,^4−6^ and vegetable oils^7,8^ can be used for the manufacture of a wide variety of sustainable materials and products, including elastomers, plastics, resins, and coatings. There is increasing interest in using biomass and lignocellulose as feedstocks,^9,10^ and of particular relevance to this paper, several bio-based acrylate resins for coating applications have been prepared.^11^ Not only are these coatings favorable for environmental reasons, but bio-based acrylates have also been shown to have superior properties to their oil-based counterparts.^12^

**Figure 1:**
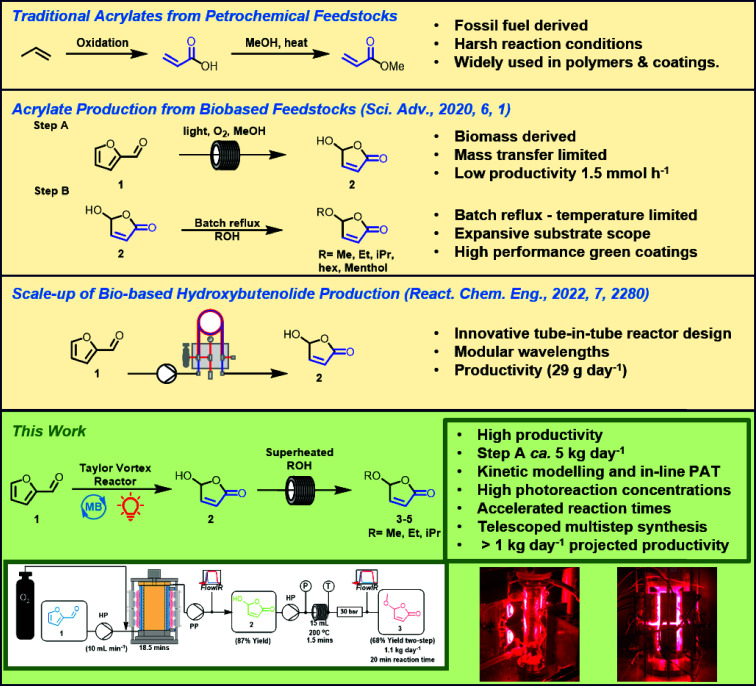
The journey from traditional acrylates (from petroleum feedstock) to furfural- and biomass-derived monomers, highlighting the technical innovations by Hermens et al. using methylene blue as photosensitizer^20^ and our further upscaling of this process described in this paper.

Furfural (**1**) is a common feedstock derived from lignocellulosic biomass, which has been used to produce bio-based acrylate monomers and has recently attracted interest as a platform chemical due to both its versatility^13^ and low cost.^14^ The application of alkoxybutenolide monomers, derived from furfural, as high-performance coatings was recently demonstrated, and their durability was tested.^15,16^ Furfural undergoes a [4 + 2] cycloaddition with photochemically generated singlet oxygen (^1^O_2_) and forms γ-hydroxybutenolide **2** in the presence of methanol, which can be followed by a thermal condensation reaction in the presence of a suitable alcohol to form the desired alkoxybutenolide monomer **3** (Figure 1).^17^ This process has potential as a bio-derived replacement for acrylate monomers in the coatings market in order to avoid reliance on oil-derived hydrocarbons.^18^ In this paper, we focus on new approaches for scaling up the consecutive reaction steps to >1 kg day^–1^ as a further demonstration of the potential of bio-based feedstocks and to readily access quantities of monomers for advanced coatings testing.

The photo-oxidation step was the limiting step in terms of batch chemical processing because batch photo-oxidation is difficult to scale due to light penetration^19^ and oxygen mass transfer from gas to solution. Continuous flow photo-oxidation provides an attractive alternative for addressing these issues, particularly light penetration, but scaling up photo-oxidation reactions using classical tubular flow reactors results in segmented gas–liquid flow, and the resulting poor mass transfer can hinder scale-up.^20^ There have been several examples of innovative flow reactor designs that enhance multiphase mass transfer using membranes to deliver gas microbubbles.^21,22^ Other designs such as the continuous spinning disk^23^ and the continuous Taylor Vortex reactor^24,25^ were developed with active mixing using a rotor–stator design, generating efficient multiphase mass transfer and often resulting in reduced reaction times in a process platform that largely decouples the mixing from the residence time.

The batch condensation was previously reported using ambient-pressure batch conditions which were dictated by the solvent selection, and scaling up these thermal transformations can be problematic with a large manufacturing footprint and the associated problems with heat transfer.^26^ Flow chemistry enables access to an expanded process window by superheating classical organic solvents above their boiling points with little reactor modification required.^27−32^ Superheated flow chemistry has also enabled the removal of harsh acid catalysts needed to overcome the activation energy of some processes.^33^ There is increased activity in developing linked flow reactions into telescoped sequences since such operation has a number of advantages, including increasing the efficiency of the process and potentially minimizing/avoiding handling of hazardous intermediates.^34^

In this work, we investigated the PhotoVortex reactor for the intensification and scale-up of the photo-oxidation of **1** with ^1^O_2_ and undertook the first flow synthesis of **3**–**5** using superheated reaction solvents to accelerate the reaction. We also carried out a preliminary study toward the synthesis of **3** as a telescoped multistep flow process with a potentially significant reduction in solvent waste by eliminating intermediate isolation steps. (These experiments require careful risk assessment—see the Safety Note at the end of this paper.)

## Results and Discussion

### Photo-oxidation in a “PhotoVap” Reactor

**Figure 2:**
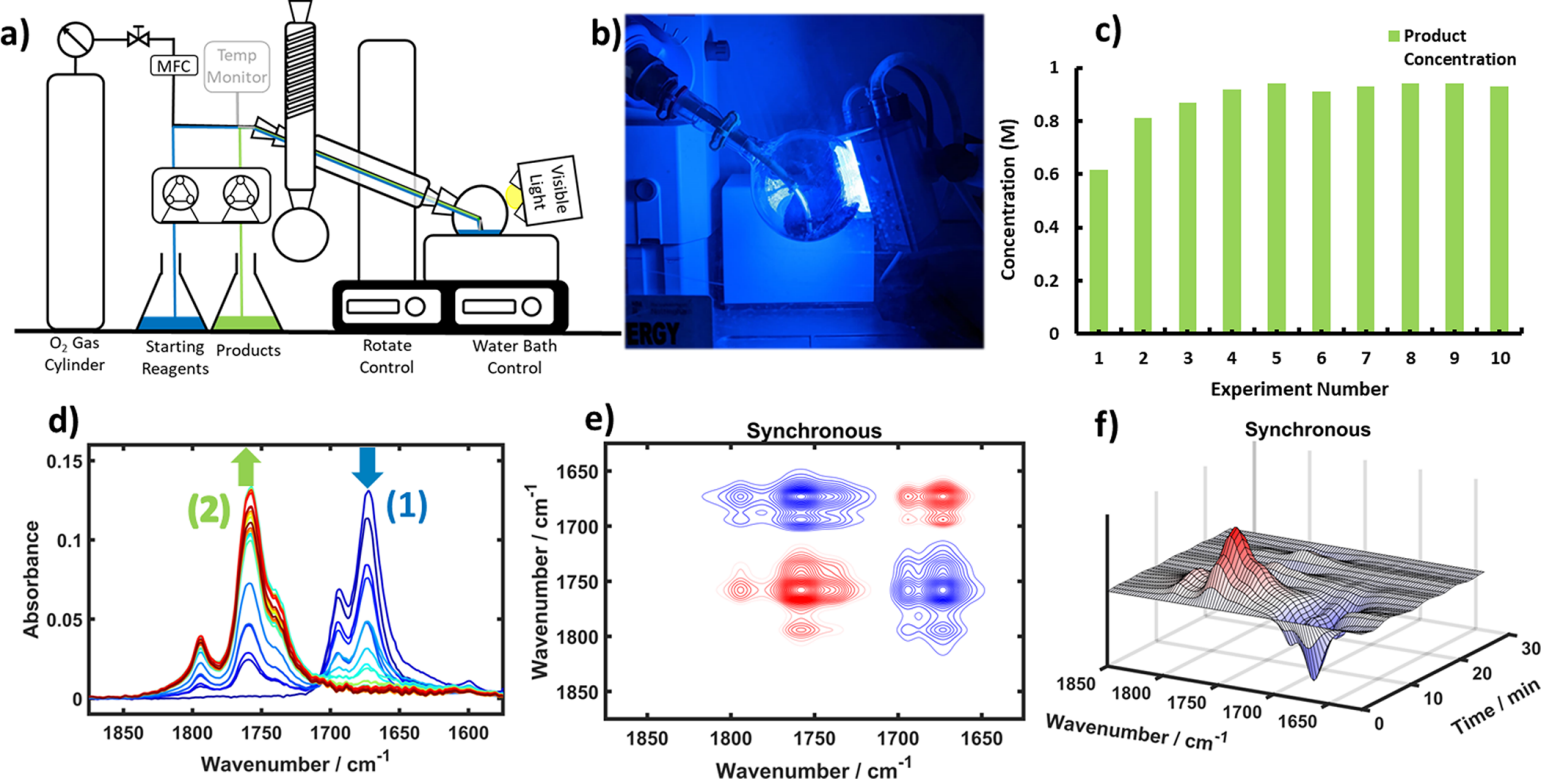
(a) Schematic of the PhotoVap reactor operating in semicontinuous mode. The flow of the dissolved aqueous O_2_ is controlled *via* a mass flow controller (MFC) and the peristaltic pumps pumping solutions in and out of the reactor at set time intervals (controlled by an Arduino board). Reaction temperature was monitored *via* a Type K thermocouple which sits at the bottom of the reaction flask. (b) Photo of the PhotoVap in operation (taken through a blue filter with the cooling bath removed for the photo). (c) Plot of the product concentration stabilizing over the course of 3.5 h of semicontinuous experiment (10 cycles) with an irradiation time of 20 min; conversions and yields were monitored by NMR. (d) Off-line IR measurements showing the consumption of **1** and formation of **2** over time. (e) Synchronous 2D-COS enabled the visualization of the conversion of **1** to **2** as well as the absence of methyl formate coproduct evident in the negatively correlated cross-peaks. (f) Synchronous PCMW2D of the off-line IR spectra. Here the perturbation is the reaction time, and peaks of opposite sign correlate negatively at each slice of the time axis, allowing for clear observation of the reaction proceeding and the respective rates of this process for the conversion of **1** → **2**. Again, there is a clear absence of a methyl formate peak.

Preliminary studies were carried out on the photo-oxidation of furfural (**1** → **2** in Figure 1) using a modified rotatory evaporator (PhotoVap).^35^ The PhotoVap generates a thin film of reaction solution that acts as a large surface over which the light can penetrate and O_2_ can transfer into the solution (see Figure 2). A similar reactor was used in the previous report,^20^ but our approach differs in two principal ways: (i) a computer-controlled Arduino board automates the semi-continuous operation of the evaporator and two pumps, which can be programmed to charge fresh aliquots of starting mixture, and (ii) the LEDs are multiwavelength rather than monochromatic, which enables a range of different photosensitizers to be used without any need to change the LEDs. This arrangement allows irradiation of the thin film for a fixed amount of time before removal and replacement of the solution with a fresh aliquot.

Figure 2a depicts a schematic of the PhotoVap. This arrangement proved to be a stable and robust setup, with batches being processed reproducibly under full computer control. Previously, a productivity of 30 mmol h^–1^ was reported for the desired hydroxyfuranone **2** in batch mode with eight 100 W LEDs, with quantitative yields when a positive pressure of O_2_ was supplied to the system *via* a balloon.^20^ In our experiments, we enhanced gas–liquid mass transfer by bubbling the O_2_ gas through the pool of liquid at the bottom of the rotating flask. Initial experiments were undertaken without cooling, and the progress of the reaction was monitored by off-line FTIR (Figure 2d). It was clear that the conversion of starting material to product can easily be monitored, but there was an absence of strong signals from the formation of the expected byproduct, methyl formate (1732 cm^–1^). The absence of methyl formate in the FTIR spectrum can be visualized by 2D correlation spectroscopy (2D-COS),^36^ with missing cross-peaks in the synchronous correlation spectrum, along with its absence from the synchronous perturbation correlation moving window 2D analysis (PCMW2D). PCMW2D is particularly useful in reaction monitoring, as it allows for the determination of how the spectral variation depends on the perturbation applied, in this case the reaction time; however, the perturbation could also be in the form of adjustments to the flow conditions, such as temperature or pressure. The result is consistent with the methyl formate (bp 32 °C) being lost by evaporation under these conditions; subsequent experiments in the PhotoVap were undertaken with active cooling to *ca.* 15 °C. As shown in experiments with the Vortex reactor below, the presence of methyl formate does not appear to affect the outcome of this reaction, but its uncontrolled loss through evaporation could present safety problems if the reaction were carried out on a larger scale. The PhotoVap was operated for 3.5 h (10 cycles) with an irradiation time of 20 min per cycle, and this gave high conversions and yields (92%). Initial product concentrations were lower than might be expected, but this was only due to a dilution effect, which is commonly seen in flow chemistry when reactors or outlet pumps have been primed with pure solvent at the start of the experiment. The conversion stabilized after four cycles. Despite using lower-power LEDs (360 W) than in the previous report, we achieved 28 mmol h^–1^ with less than half the power, indicating that gas–liquid contact is a critical factor toward efficient photo-oxidation reactions. Taking the PhotoVap forward, however, raises potential safety limitations regarding the scale-up of the reaction/reactor with bubbling O_2_ into the reaction solution in the presence of large free volumes of gaseous O_2_.

### Photo-oxidation in the Small Taylor Vortex Reactor

**Figure 3:**
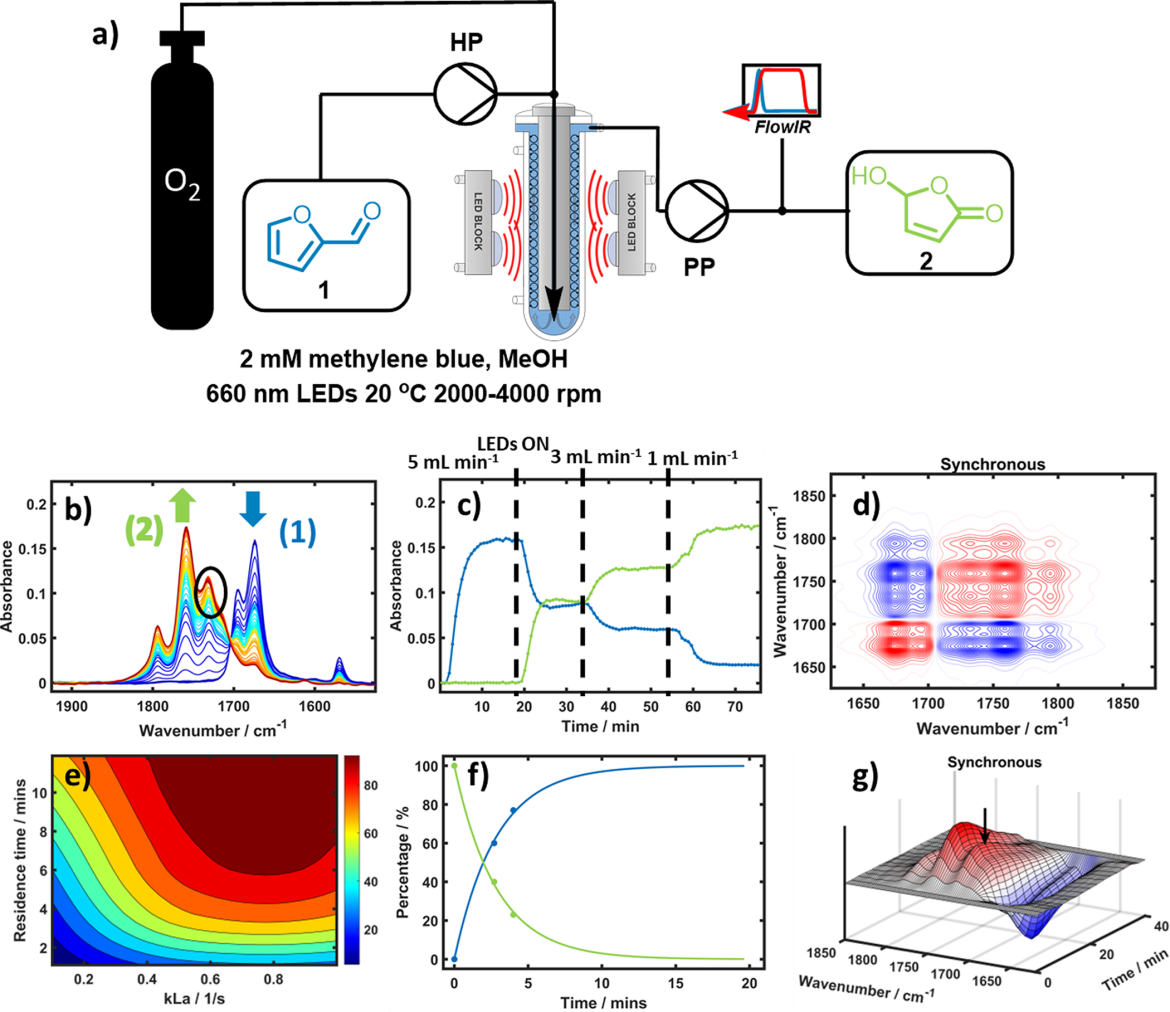
(a) Schematic of the photochemical vortex reactor, including in-line FTIR analysis at the outlet of the reactor. HP is the HPLC pump, and PP is the peristaltic pump. (b) ReactIR spectra from 1900 to 1600 cm^–1^ used to monitor the photochemical oxidation of **1** in MeOH solution at 20 °C. Spectra are colored blue (start of reaction) to red (end of reaction); methyl formate was observed at *ca.* 1732 cm^–1^ in the black circle. (c) Monitoring of consumption of **1** (blue) and formation of **2** (green) at decreasing flow rates (5, 3, and 1 mL min^–1^) showing the positive effect of increased residence time on product formation. (d) Synchronous 2D-COS shows formation of methyl formate by clear cross-peaks at *ca.* 1732 cm^–1^ as well as the conversion of **1** to **2**. (e) Contour plots were generated with Reaction Lab software showing the influence of residence time and mass transfer coefficient, *k*_L_*a*, on the yield of **2**. (f) Plot of the Reaction Lab model versus experimental data points showing the good fit of the model. (g) Synchronous PCMW2D of the real-time IR spectra. Again, the perturbation is the reaction time and allows for the observation of the reaction proceeding; however, in this case the methyl formate peak is clearly visible and is concomitant with the product formation. In some experiments, modest photobleaching of methylene blue was observed.

There have been several reports of photogeneration of singlet oxygen in flow systems,^37−39^ and we have recently reported the use of Taylor Vortex reactors for both continuous photochemistry^24,25^ and electrochemistry.^40,41^ In addition to our initial publications, further reports using Vortex reactors for photochemistry^42,43^ have been published during the review process for this paper. The photochemical version of our reactor, the PhotoVortex, ensures highly efficient gas–liquid micromixing, which enables lower equivalents of gaseous O_2_ to be used, with very small volumes of gaseous O_2_ for generating singlet oxygen. Most of our experiments are designed to be near-stoichiometric so that during periods of long-term operation >85% of the introduced O_2_ gas is completely consumed by the reaction. As used here, the smaller PhotoVortex Reactor consists of a transparent Pyrex-jacketed tube that is sealed at the bottom and contains a polished 316 stainless steel cylindrical rotor with a narrow bore running coaxially through its center. We initially used our smaller PhotoVortex with a 1 mm annular gap size between the rotor and the glass tube, giving an irradiated volume of *ca.* 8 mL. The PhotoVortex used in this paper was modified slightly from our previous reports^24,25^ by using newly designed LED blocks (3 × 200 W blocks) equipped with lenses which act both to focus the LED output and to protect the LED chips. The output wavelength of these LEDs (660 nm) overlaps with the absorption band of methylene blue in methanol (λ_max_ = 654 nm). In these experiments, the O_2_ gas was dosed *via* a T-junction at the reactor inlet as shown in Figure 3a. Our publication^25^ discusses the Taylor numbers of this reactor and the scaled-up Vortex reactor described below.

Building upon the off-line IR measurements used in the experiments in the PhotoVap above, we employed real-time IR reaction monitoring with a custom-made flow cell and quantitative IR spectra monitoring. The IR data were converted to concentration *via* a series of product calibration standards and using multivariate curve resolution (MCR) and partial least-squares (PLS) models. Initial reaction conditions were obtained from the literature, and a methylene blue concentration of 2 mM was chosen due to the inner-filter effects observed by Hermens et al.^20^ For proof of concept with the new LEDs, the reaction was carried out with 0.5 M **1**, and the amount of O_2_ was initially varied between 1.05 and 1.2 equiv. Entry 1 in Table 1 shows that the reaction proceeded under these conditions with a high efficiency. The concentration was then doubled to match the PhotoVap experiments, and Table 1, entry 2 shows an equally efficient conversion and yield compared to the previous experiments using the PhotoVap despite reduced reaction times of 8 min. This is consistent with the more efficient mass transfer we have observed in the PhotoVortex.

**Table 1:**
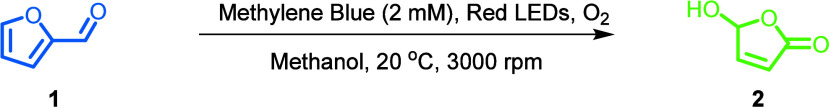
Optimization Table for the Photo-oxidation of Furfural in the Vortex Reactor 0.5–2 M, Highlighting the Trade-Off between High Yield and High Productivity

entry	[**1**] (M)	space time (min)^a^	equiv of O_2_	conv. of **1** (%)^b^	yield of **2** (%)^b^	productivity (g day^–1^)^c^
1	0.5	8	1.2	95	92	66
**2**	**1**	**8**	**1.2**	**98**	**95**	**140**
3	1	4.	1.2	95	77	220
4	1	2.7	1.2	87	60	260
5	1	8	2.5	93	88	130
6	1	4	2.5	85	80	230
7	1	2.7	2.5	74	69	300
8	1	16	2.5	98	96	70^d^
**9**	**1.5**	**2.7**	**2.5**	**75**	**64**	**410**
10	2	2.7	2^e^	55	46	397

a “Space time” refers to the residence time in the irradiated volume of the reactor (which is smaller than the total reactor volume). It is calculated by dividing the irradiated volume by the flow rate of the reaction mixture.

b NMR yields calculated using 1,3,5-trimethoxybenzene as the internal standard.

c Projected productivity over 24 h of operation.

d Coloring of the glass (fouling) was observed.

e Flow rate limit of the mass flow controller.

Figure 3c shows preliminary results for the in-line FTIR reaction monitoring of the consumption of furfural and formation of 5-hydroxyfuranone at different flow rates (5, 3, and 1 mL min^–1^) using a rotor speed of 3000 rpm. As expected, increasing the residence time can be used to increase product formation. Apart from the concentration and therefore yield provided using our MCR model, the FTIR data permitted the identification of the point when steady-state operation had been re-established following a change in reaction conditions. We were then able to shorten our optimization time using in-line FTIR by using the steady-state readout, which was observed after approximately three space times (*i.e.*, the time taken for the solution in the irradiated volume to be changed three times), rather than waiting much longer for three system volumes of reaction mixture to pass through the entire reactor assembly (*i.e.*, an amount of solution equal to 3 times the volume of the whole flow system). In these experiments, methyl formate IR bands are clearly observed by in-line monitoring (Figure 3b) and are even more striking in the 2D-COS analysis, with clear cross-peaks and correlation peaks (Figure 3d,g). This contrasts with the initial experiment in the PhotoVap (Figure 2e,f).

We generated a crude kinetic model for the photo-oxidation of **1** to **2** under our reaction conditions using the commercially available software package Reaction Lab.^44^ Our aim was to use this software to reduce the number of experiments required to obtain purposeful information from the model. In particular, we were interested in learning how well a kinetic model could predict the reactor performance based on reaction profiles determined from very limited amounts of experimental data. Once the initial model had been generated, further data points could be added to improve the model in an iterative process. In the Reaction Lab model, we defined the irradiated reactor volume as the overall volume and defined an individual scenario for each set of starting conditions (*e.g.*, 2-furfural concentration, O_2_ pressure, rotation speed/*k*_L_*a*). Each experimental data point was determined by running the reaction at different flow rates. The general methodology for collecting reaction profiles from a flow reactor for kinetic modeling has been described previously.^45^ The workflow for completing a Reaction Lab model can be found in the Supporting Information.

Briefly, we started by taking two data points from our initial experiments (Table 1, entries 3 and 4) with a rotation speed of 3000 rpm. Photon flux was calculated with calibrated Ocean Optics fibers in “Absolute Irradiance” mode. However, the most important assumption that we found to affect the model was the mass transfer coefficient (*k*_L_*a*), and this value was set at 1 s^–1^, which we used to indicate a relatively well mixed gas–liquid system. At high *k*_L_*a* values on this order of magnitude, mass transfer no longer has any impact on the rate of the overall process, as the system remains O_2_-saturated throughout. The contour plot generated with Reaction Lab software, shown in Figure 3e, illustrates the influence of *k*_L_*a* and the residence time on the yield of **2**. The preliminary model fitted the initial data well (Figure 3f) and found that the rate-determining step was the reaction of photochemically generated ^1^O_2_ with **1**. An optimization was then carried out with an objective target function of 95% yield, and the model predicted that this would occur with a residence time of 8.5 min.

Using this initial model, we investigated the performance of the PhotoVortex reactor by obtaining experimental results for three different rotation speeds (0, 1000, and 3000 rpm) and carrying out a simulation with a fixed liquid flow rate of 1 mL min^–1^ and 1.2 equiv of O_2_. Reaction Lab was able to display the influence of rotation speed at a given residence time (end time in Reaction Lab output), providing an output of predicted *k*_L_*a*. Experimentally, with no rotation, a yield of 22% was observed, corresponding to an estimated *k*_L_*a* value of 0.15 s^–1^. As the rotation speed increased to 1000 and 3000 rpm, the formation of Taylor vortices could clearly be observed through the transparent walls of the reactor, and yields of 68% and 95% were obtained, corresponding to *k*_L_*a* values of 0.3 and >0.5 s^–1^, respectively. Positively, the addition of two further experimental data points to the model improved the “Optimization” function, and for a yield of 95% an end time of 8.3 min was estimated. Model validation was achieved from the small Design of Experiments study, whereby a 95% yield was achieved with a space time of 8 min (1 mL min^–1^).

In an effort to maximize the productivity of this reaction, we aimed to increase the concentration further to 1.5 and 2 M (Table 1, entries 9 and 10), where we observed a small dropoff in yield due to the elevated oxygen flow rates required to satisfy the reaction stoichiometry. The detrimental effect on the reaction was due to instability of vortices and the resulting reactor bypass of slugs of gas. Nevertheless, reasonable yields of 64% and 46% were still achieved by increasing the equivalents of oxygen further in the cases where flow instability was already an issue (*e.g.*, Table 1, entries 4 and 7). With these conditions, a projected maximum productivity of *ca.* 400 g day^–1^ was achieved with a 64% yield in a reactor with an irradiated volume of *ca.* 8 mL or a lower productivity of 140 g day^–1^ but with a much higher 95% yield (3 and 1 mL min^–1^, respectively, and 3000 rpm). During these experiments, we did not attempt to change the way in which O_2_ was fed into the reactor.

### Intensification of the Thermal Condensation of Hydroxyfuranone to Alkoxybutenolides

**Figure 4:**
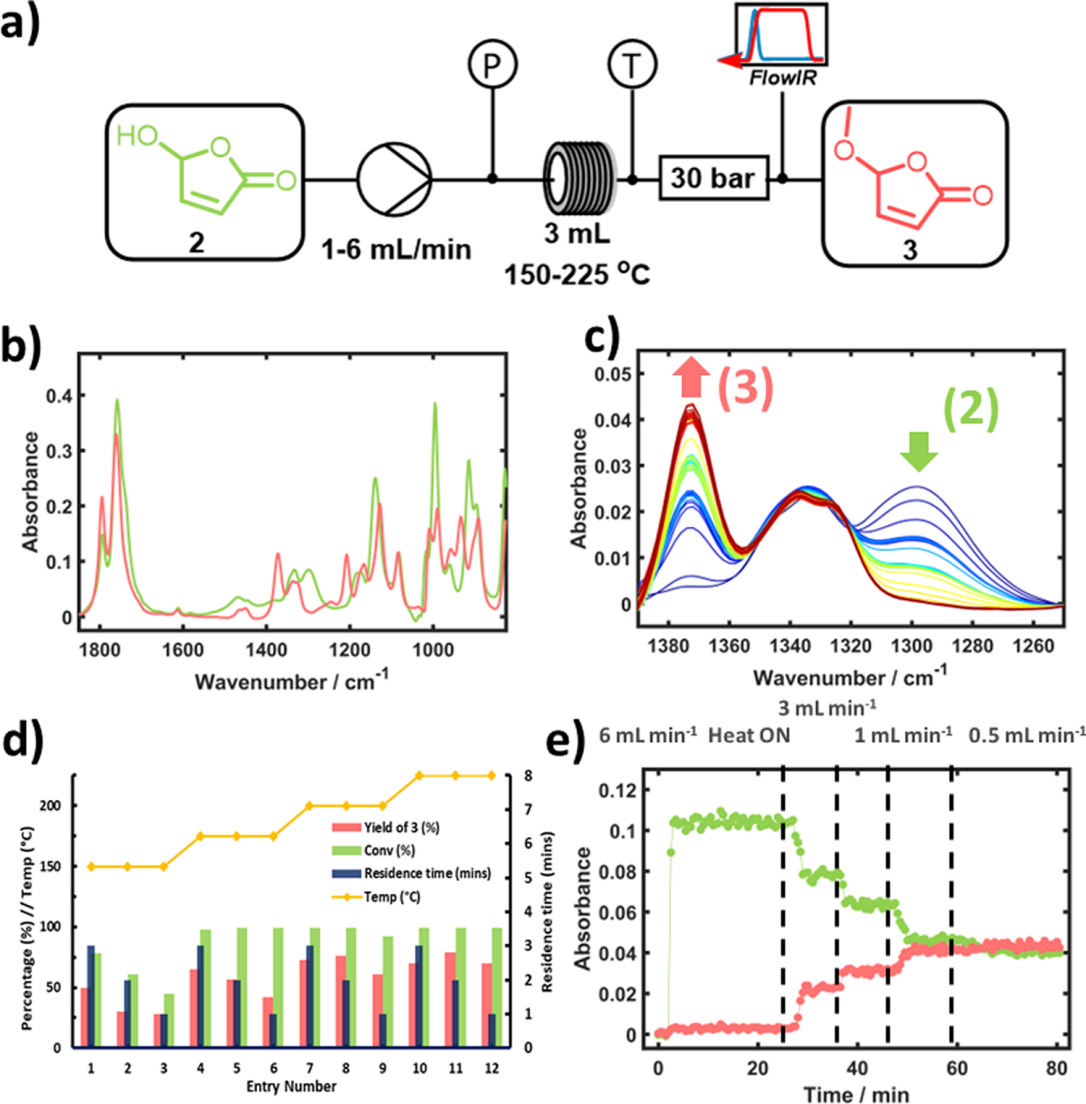
(a) Simplified process schematic for the thermal condensation reaction in a stainless steel tubular reactor. P is the pressure gauge, and T is the thermocouple. (b) FTIR spectra of the pure individual components 5-hydroxyfuranone (**2**) (green) and 5-methoxybutenolide (**3**) (rose). (c) Zoomed section of a series of ATR-FTIR spectra showing how the consumption of **2** and formation of **3** can be easily monitored. (d) Bar chart of the data from initial experiments at 0.1 M, highlighting the influence of temperature on reaction yield. Entries 7 and 8 show increased yields despite shorter residence times. The poor mass balance at higher temperatures is attributed to polymerization of the butenolide product. (Note that the data in Table 2 are for a higher concentration of 1 M rather than the concentration of 0.1 M in this figure.) (e) FTIR data were recorded at a reaction temperature of 150 °C, showing the effect of different flow rates on the consumption of **2** (green) and formation of **3** (rose).

In this section, we investigate how the thermal condensation reaction of **2** with MeOH could be sufficiently accelerated in a high-temperature tubular reactor to match the productivity of the continuous photo-oxidation step in the PhotoVortex reactor obtained above. Long reaction times of *ca.* 20 h under reflux conditions have previously been reported in batch,^20^ which is problematic for the integration into a two-step (photochemical + thermal) continuous process. Therefore, we exploited the expanded process window available in pressurized continuous flow in order to accelerate the conversion of **2** to **3**, reducing the reaction time for this second step while maintaining a small reactor footprint (Figure 4a).

Initial reactions were carried out at 0.1 M in a 3 mL stainless steel tubular reactor, and we investigated the relationship between temperature and residence time on the formation of **3** while monitoring the system pressure to ensure that no precipitation or blockage in the reactor occurred. Conversions and yields were monitored *via* NMR and can be visualized in the bar plot (Figure 4d). This plot shows that as the temperature increases, a step change occurs at 200 °C and 1–2 mL min^–1^, whereby the yield begins to increase as the residence time is decreased (Table 2, entries 7 and 8).

**Table 2:**
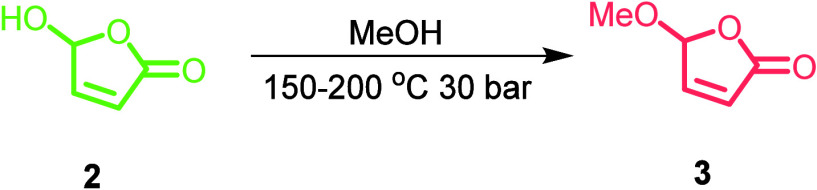
Optimization Table for Thermal Condensation of 5-Hydroxyfuranone at 1 M^a^

					productivity	
entry	temp. (°C)	residence time (min)	conv. of **2** (%)	yield of **3** (%)	g day^–1^	mmol h^–1^
1	150	3	91	72	120	43
2	150	1.5	75	61	200	73
3	150	1	68	53	260	95
4	175	3	92	79	130	47
5	175	1.5	91	84	270	100
6	175	1	86	73	360	130
7	200	3	98	80	130	48
8	200	1.5	96	82	270	100
9	200	1	95	81	400	140
10	200	0.8	92	78	510	190
11	200	0.6	89	76	630	230
12	200	0.5	86	74	730	270

a Conversion and yield were monitored by NMR with 1,3,5-trimethoxybenzene as the internal standard. The reactor volume was 3 mL. Productivity in g day^–1^ was predicted for a 24 h reaction period.

It was for this reason that we decided to limit the upper boundary reaction temperature to 200 °C while attempting to scale up this process. Once again, the application of in-line FTIR proved to be highly significant, demonstrating its effectiveness in screening and optimizing this thermal condensation (Figure 4e).

Further scale-up of the thermal condensation reaction was achieved by optimizing the balance between flow rate and temperature as the concentration of the reaction was to be increased to 1 M (Table 2). As expected, as the concentration of **2** was increased, the reaction proceeded more favorably and as a result required a lower reaction temperature to achieve acceptable yields. Productivities for this condensation matched and even exceeded those of the photo-oxidation of **1** in the PhotoVortex reactor outlined above, and at a flow rate of 6 mL min ^–1^, a projected productivity of >700 g day^–1^ was achieved in our small flow reactor (3 mL).

**Figure 5:**
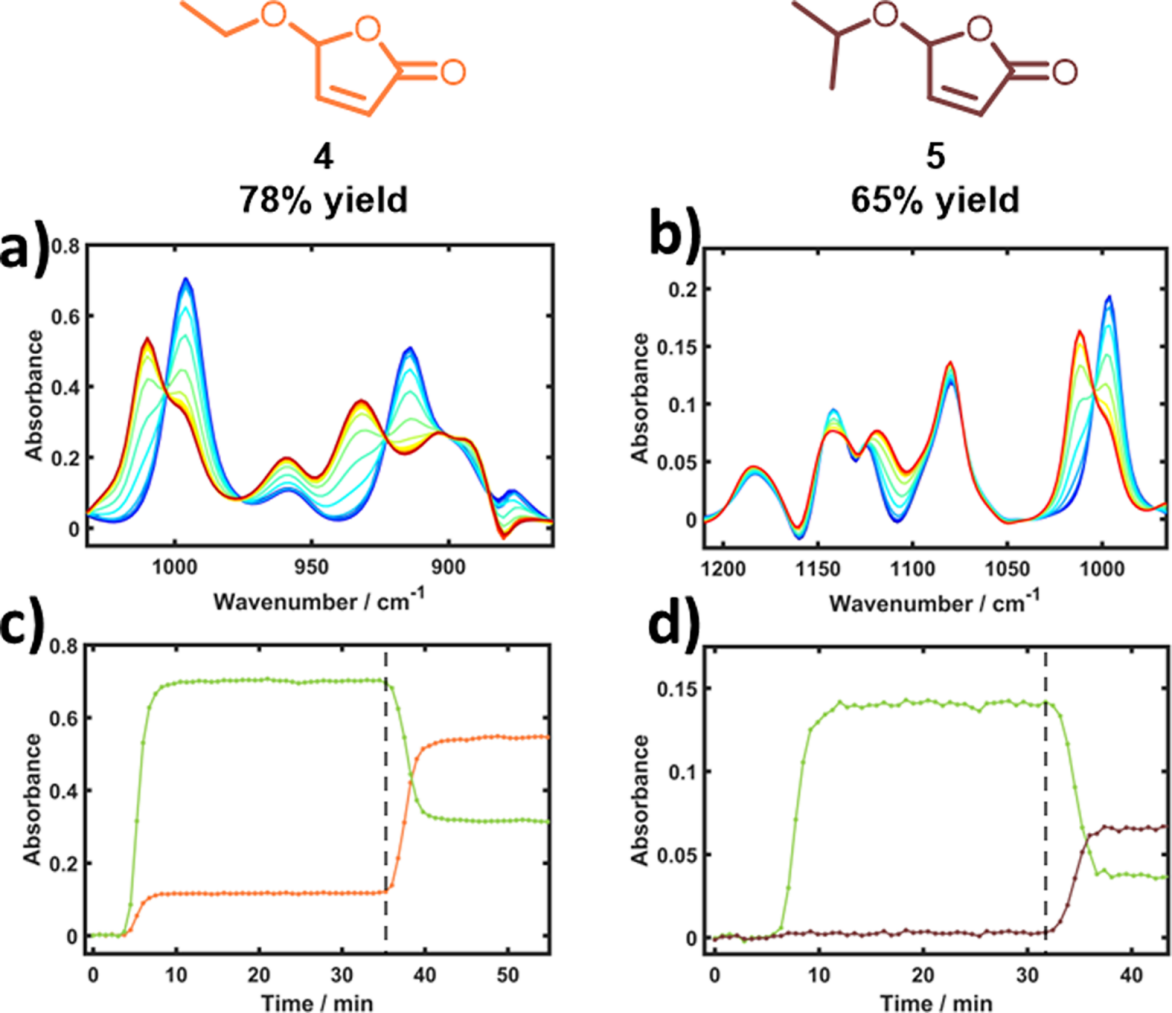
Investigation of additional solvents (EtOH and iPrOH) for the synthesis of alkoxybutenolide monomers **4** and **5**. Synthesis was carried out at 200 °C and 2 mL min^–1^ from the optimized conditions from Table 2. (a, b) Spectral region over time (blue to red). (c, d) FTIR trends showing the consumption of 5-hydroxyfuranone (green) and the formation of **4** (orange) and **5** (brown). The dashed vertical lines indicate when heating was turned on.

With the optimized reaction conditions, a small study of substrate scope was carried out by varying the reaction solvent to produce the condensation products of other alkoxy substituents on the furanone (see Figure 5), where reactions were carried out at 1 M substrate concentration, 200 °C, and 2 mL min^–1^. As the R substituent grows in bulk, the conversion drops slightly, but overall, the reaction efficiency remains similar. Facile modification of the R substituent on the alkoxybutenolide is of importance because these substituents influence the properties of coatings produced by the polymerization of the alkoxyfuranones.^20,46^

### Telescoped Production of Alkoxybutenolide **3** Using the Small-Scale Taylor Vortex Reactor

To investigate the two reaction steps outlined above in a telescoped flow synthesis, it was necessary to consider the transfer of the product from one reactor to another. We used a buffer tank because a peristaltic pump was required at the Vortex outlet due to gas–liquid segmented flow exiting the reactor. This peristaltic pump would be unable to apply suitable pressure to overcome the high back-pressure requirement to superheat methanol to 200 °C. Therefore, a second HPLC pump was used to charge the thermal reactor.

**Figure 6:**
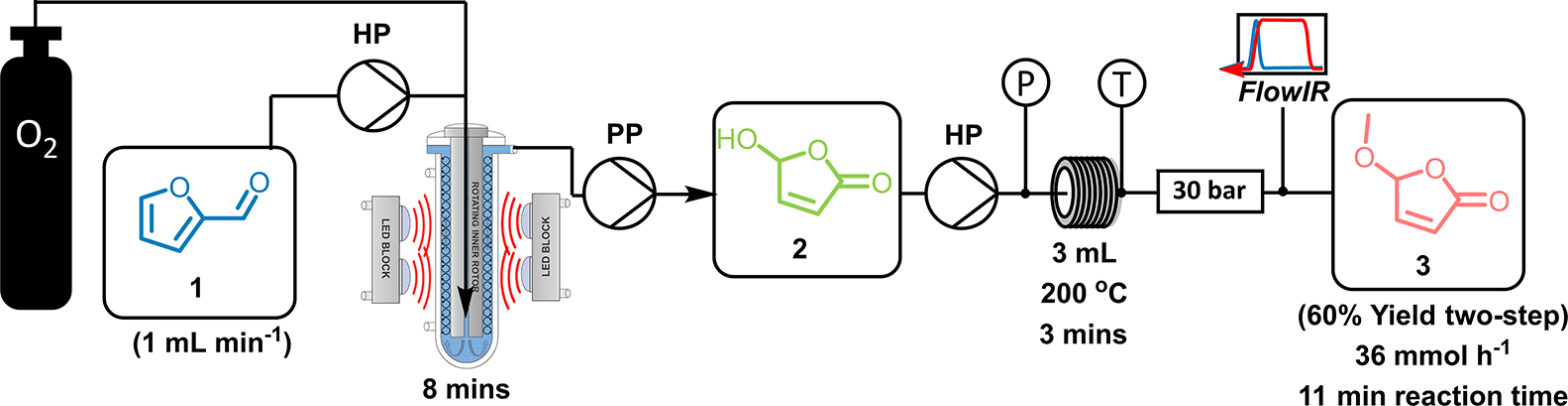
Simplified schematic of the telescoped reaction setup using a small Taylor vortex reactor. HPLC pumps are labeled HP, and the peristaltic pump is labeled PP. **2** was collected in a buffer tank before being charged into the reactor. In-line FTIR was used at the outlet of the second step to monitor both of the reactions.

Using the highest-yielding conditions from the small-scale vortex reactor, 1 mL min^–1^ and a 1 M solution of **1** were selected for the test of the telescoped reaction (Table 1, entry 2). The photo-oxidation of **1** was carried out in the small-scale vortex reactor for approximately 3 h to reach steady state and collect material in the buffer tank. The crude product stream was then pumped into the thermal reactor at a rate of 1 mL min ^–1^. A 93% NMR yield was obtained for the photochemical reaction with an overall two-step yield of 60% for methoxybutenolide **3**, which corresponds to a slightly reduced second-step yield of 65% compared to the pure-component reaction (Table 2, entry 7). Even so, this still accounts for a moderate predicted productivity of 36 mmol h^–1^ or 99 g day ^–1^ without any intermediate isolation or purification. Both reaction steps were monitored by FTIR at the outlet of the thermal reactor, as depicted in Figure 6. Nevertheless, the process was still limited by the photo-oxidation of **1**, and the high relative rate of the condensation reaction led us to reinvestigate and scale up the initial photo-oxidation.

### Photo-oxidation in the Large Taylor Vortex Reactor

The productivity of the photo-oxidation step in the small vortex reactor was limiting the productivity of the whole telescoped flow synthesis described above. Therefore, our new objective was to further scale up the photo-oxidation of **1** to better match the productivity of the thermal step. Recent advances in scale-up of photochemical reactions in terms of both chemical scope and reactor design have been reviewed.^19,47^ We used our larger-scale PhotoVortex reactor^25^ with a *ca.* 185 mL irradiated volume and a 2 mm gap width.

Initial reaction conditions were carried out with a jacket temperature of 20 °C, similar to that used with the small-scale PhotoVortex reactor. Initially, we observed poor reaction efficiencies with low yields at equivalent space times (see Table 3, entries 1 and 2). An interesting observation using those conditions was that the reactor outlet temperature increased to >40 °C and the methoxylated product **3** was formed thermally in low yields, resulting in reduced yields of the desired hydroxybutenolide product **2**. This could be due to some of the intermediate endoperoxide decomposing thermally back to **1**, as suggested by Aubry.^48^ We investigated this further using a twofold strategy: (i) lowering the chiller temperature for the reactor coolant to −5 °C and (ii) precooling the reaction mixture prior to the reactor with a salt–ice bath (*ca.* −10 °C). These interventions resulted in the feed solution entering the reactor at between 0 and 5 °C. However, the temperature of the solution at the reactor outlet was still *ca.* 30 °C. With conditions optimized, an 85% yield of **2** (+5% **3**) was achieved with a flow rate of 11.5 mL min^–1^, providing 570 mmol h^–1^ or 1.3 kg day^–1^. Higher projected productivities were obtained with flow rates of 23, 46, and 69 mL min^–1^, which afforded 1000 mmol h^–1^ (2.4 kg day ^–1^), 1430 mmol h^–1^ (3.4 kg day^–1^), and 1660 mmol h^–1^ (4.0 kg day^–1^), respectively (Table 3, entries 4–7).

**Table 3:**
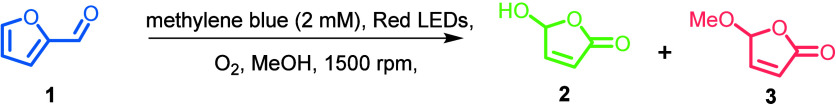
Optimization Table for Large-Scale PhotoVortex Photo-oxidation of **1** at 1 M^a^

entry	chiller temp. (°C)	space time (min)	yield of **2** (%)^c^	yield of **3** (%)^c^	productivity of **2** (kg day^–1^)^d^	space-time yield (mol h^–1^ mL^–1^)
1	20	8	55	5	1.8	4.1
2	20	4	44	2	2.9	6.6
3	–5	8	61	4	2.0	4.6
4	–5^b^	8	73	2	2.4	5.4
5	–5^b^	16	85	5	1.3	3.2
6	–5^b^	4	52	1	3.4	7.8
7	–5^b^	2.7	40	trace	4.0	9.0

a Reactions were typically run for 3 h. This is shorter than the time reported for deposits forming on the reactor walls (fouling) in a different design of The PhotoVortex reactor.^42^ However, we normally ran several sequential experiments in *our* reactor over a period of a week without any intermediate cleaning of the glassware (apart from flushing with clean solvent between experiments), and we did not observe any significant fouling. The choice of rotation speed was ultimately empirical, but formation of the Taylor vortices depends on the surface velocity of the rotor rather than its rate of rotation. Therefore, the large Vortex reactor can be rotated more slowly than the small Vortex reactor without deterioration of the mixing, as previously discussed when the Vortex reactor was first scaled-up.^25^

b The feedstock and precooling loop were cooled to −10 °C.

c ^1^H NMR yields with 1,3,5-trimethoxybenzene as the internal standard.

d Predicted productivity over a 24 h reaction period.

### Kilo-Scale Telescoped Flow Synthesis of **3**

**Figure 7:**
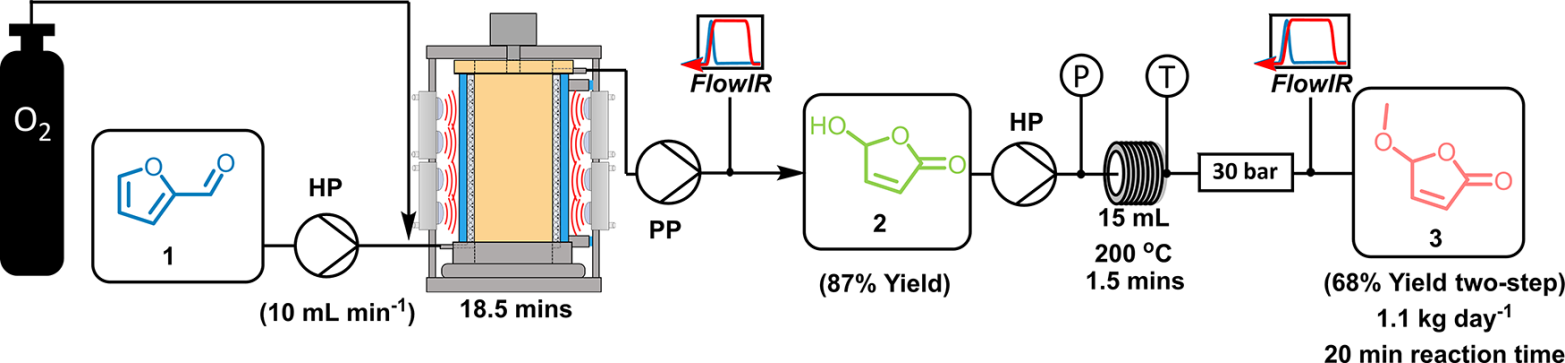
Simplified schematic of the scale-up of the telescoped reaction steps toward the synthesis of methoxybutenolide **3** using our larger Vortex reactor (as described in the text). Pumps are labeled as HP for the HPLC pump and PP for the peristaltic pump. **2** was collected in a buffer tank before being pumped into the stainless steel tubing reactor. Pressure and temperature monitors and trips (P and T) were used in-line to ensure safe operation. In-line FTIR was also used to ensure that a steady state was achieved and showed that the reactions were both stable and robust.

Using the conditions from the telescoped process in the smaller-scale Vortex setup, we used the output stream of the large vortex reactor at 10 mL min^–1^ for a proof-of-concept experiment, as shown in Figure 7. A 15 mL thermal reactor was used, consisting of coiled 1/8″ stainless steel tubing to maintain plug flow properties similar to the 3 mL reactor used previously. This resulted in the condensation having a slightly shorter residence time compared to the small-scale telescoped process. Nevertheless, a reaction temperature of 200 °C was chosen for our investigation. The other conditions were as described in Table 3, with the jacket temperature of the large-scale vortex reactor set to −5 °C and the feedstock and cooling loop set between −5 and −10 °C. An 87% yield was achieved from the photo-oxidation in the large vortex reactor at 10 mL min ^–1^ with a 400 mL min^–1^ flow rate of O_2_. This photo-oxidation product solution was fed directly into the scaled-up thermal reactor, where an overall two-step yield of 68% was achieved, with a two-step projected productivity of 1.1 kg day^–1^ being obtained for this telescoped reaction process without any need for intermediate isolation/purification.

## Conclusion

The reaction steps toward the synthesis of high-performance greener coatings were successfully scaled from 0.03 mol h^–1^ as a thin-film batch process to greater than 1.7 mol h^–1^ utilizing a fully continuous PhotoVortex. This equates to a projected productivity of 4 kg day^–1^ in a 24 h reaction period. This is a thousand-fold increase in productivity compared to 0.0015 mol h^–1^ in the single tubular FEP flow reactor setup which was originally used.^20^ We were limited, however, by the distribution of the high flow rates of gas required at increased reaction concentrations, which resulted in unstable vortices and reduced reaction yields. We are currently investigating the development of a high-pressure PhotoVortex, which should further enhance the photo-oxidation reactions by increasing the solubility of oxygen but also reducing the flow rate of gas required to satisfy the equivalents of more concentrated reactions. The formation of alkoxybutenolide monomers was accelerated from a 20 h reflux and scaled to greater than 0.26 mol h^–1^ in a flow reactor of 3 mL volume, which equates to 732 g day^–1^ for a 24 h reaction period with residence times of less than 1 min. This was simply further scaled by increasing the length of tubing, which was demonstrated in the scaled telescoped reaction process. Initial tests toward the telescoped reaction scale-up were also investigated, which yielded a 0.4 mol h^–1^ or 1.1 kg day^–1^ productivity of the methoxybutenolide without any intermediate purification steps, demonstrating viable procedures for ready access to multigram amounts of bio-based monomers for future coating studies.

## Experimental Section

### General Information

All solvents and reagents were used as obtained without any further purification, unless stated otherwise. Furfural was distilled under a vacuum prior to use. ^1^H and ^13^C NMR spectra (Bruker DPX300, 400 and 100 MHz) were recorded at ambient temperatures unless otherwise specified. Chemical shift values are reported in parts per million, and solvent resonances were used as internal standards (CHCl_3_: δ = 7.26 ppm for ^1^H and 77.16 ppm for ^13^C). For the yield/conversion, 1,3,5-trimethoxybenzene was used as an internal standard. The spectra were assigned by comparing observed chemical shifts to existing literature values. Column chromatography was carried out using an autocolumn (Teledyne ISCO Next-Gen 300+) with UV (254 nm) or ELS detection. In-line IR analysis was obtained using a Mettler Toledo ReactIR 702L with a 6.3 mm AgX DiComp. All FT-IR spectra were obtained with an air background and were then normalized to the MeOH solvent peak. O_2_ was dosed into the reactors using Bronkhorst mass flow controllers (MFCs) at ambient pressure.

### General Reactor Operation: Small and Large PhotoVortex

The recirculating chiller for cooling the LEDs was turned on and set to 10 °C, while the reactor chiller was set to the appropriate temperature for the reaction. The chillers were left to equilibrate for *ca.* 20 min. The reaction mixture was prepared as described below, after which the inlet tubing for the HPLC pump was fed into the solution and the pump was primed. The oxygen cylinder was opened and set to the desired pressure (*ca.* 1 bar). The desired flow rate was set on the MFC, and gas was dosed into the system at a low flow rate (*ca.* 5 mL min^–1^). The desired liquid flow rate was set on the inlet pump (HPLC or peristaltic), and the solution was pumped into the reactor. The second peristaltic pump for removing the products from the reactor was set to a flow rate that was suitably high to pump out the solution from the reactor. The rotation motor of the Vortex reactor was turned on, and the speed was slowly increased until the desired rotation speed was achieved. Then the LEDs were turned on at full brightness. The outlet of the peristaltic pump could then be fed into in-line FTIR whereby the steady state could be monitored and real-time concentrations obtained. If FTIR was not used, two full system volumes were allowed to pass before taking any sample for analysis; this ensured that the reactor had reached a steady state (confirmed by off-line sampling). Once the operation was complete, the LEDs were turned off, and the reactor was flushed with pure solvent at increased flow rates to clean the reactor. The inlet pump was turned off, and the rotation speed of the motor was slowly decreased to 0 rpm. The inlet pipe was switched to a container of a compatible solvent (usually the reaction solvent), and the reactor was flushed. The rotation speed was reset during this time to ensure that all of the material was removed from the reactor. Once the reactor was clean, the motor, pumps, and MFCs were turned off, and the cylinder was isolated. The recirculating chillers were also turned off provided that the LED blocks were not excessively hot.

### General Reaction Conditions: Photo-oxidation of **1** in the PhotoVap

Freshly distilled furfural (60 °C, 1 × 10^–2^ bar) was weighed into an appropriately sized volumetric flask for the desired number of experiments. Methylene blue was added to the volumetric flask, which was topped up with methanol, giving the desired catalyst concentration. The solution was homogenized and transferred to a vessel that acted as the feedstock reservoir. If operating semicontinuously, the pumps were connected to the Arduino controller, and the code for the experiments was generated by modifying serial signal times for the desired application. The MFC was set to bubble O_2_ gas into the reactor at 20 mL min^–1^, and the operating Arduino program was initiated. Samples were collected at the outlet pump at the end of each program.

When not operating in semicontinuous mode, the reactor (1 L round-bottom flask) was charged with the desired volume of the reaction mixture (*ca.* 10 mL). The rotation and illumination were manually controlled, and the reaction was carried out for the desired amount of time before the reaction flask was replaced manually. The products were analyzed by ^1^H NMR with 1,3,5-trimethoxybenzene as an internal standard.

### General Reaction Conditions: Photo-oxidation of **1** in the Taylor Vortex Reactor

The furfural and methylene blue solution was prepared as described above for the PhotoVap. The flow rate of O_2_ was calculated using the ideal gas law, and the MFC was set to the desired set point. The solution of **1** was then pumped into the system for irradiation. After irradiation for the specified amount of time, conditions were changed, or the reactor was flushed with solvent for cleaning. The products were analyzed by ^1^H NMR with 1,3,5-trimethoxybenzene as an internal standard. Methylene blue was removed by trapping with activated charcoal and filtration through Celite. For lower-yielding experiments (<75%), unreacted furfural was removed *via* column chromatography (silica gel, 90:10 *n*-pentane/ethyl acetate) before the product was recrystallized from CHCl_3_ by seeding. For higher-yielding experiments, the product could be directly recrystallized by seeding a supersaturated solution and cooling it in the freezer (−20°) before filtering to collect product **2** as a white solid.

### General Reaction Conditions: Thermal Condensation of **2** with R–OH to Form the Corresponding Alkoxybutenolide

A solution of **2** (0.1–1 M) was prepared in an appropriately sized volumetric flask with the desired alcohol solvent. The solution was homogenized and transferred to an HPLC pump for priming. Initially pure solvent was pumped through the reactor, and the back-pressure regulator was set to the required pressure set point. The reactor was heated to the desired temperature, and a valve was opened to allow the reaction solution to be pumped into the reactor. Samples were taken once steady state was achieved, and reaction conditions (temperature or flow rate) were changed when screening was the objective. When the experiment was completed, the reactor was flushed with pure solvent to clean the system, after which the temperature was reduced to ambient and the back pressure was released. The product yields were analyzed by ^1^H NMR with 1,3,5-trimethoxybenzene as an internal standard. Alkoxybutenolide isolation was carried out *via* column chromatography (silica gel, 90:10 *n*-pentane/ethyl acetate) to purify the products **3**–**5** as colorless oils.

### General Reaction Conditions: Telescoped Flow Synthesis

The reaction mixture of **1** and methylene blue was prepared as before, and the O_2_ gas flow rate was set as above. The solution of **1** was then pumped into the system for irradiation. Once steady state was reached, the output of the photoreactor was pumped into a buffer tank, where it was collected before being pumped into the pressurized thermal reactor. The yield of **2** was calculated by analyzing the contents of the buffer tank using 1,3,5-trimethoxybenzene as an internal standard. The inlet pump (HPLC) for the thermal reaction was primed, and the solution of **2** was pumped from the buffer tank into the thermal reactor. The back-pressure regulator and the heating block were set to their desired set points. The products were collected in an appropriately sized waste container where the final product, **3**, was collected. The overall yield of the two-step process was calculated by using ^1^H NMR with 1,3,5-trimethoxybenzene as the internal standard. The PhotoVortex and thermal reactors were cleaned separately following the general procedures outlined above.

### *Safety Note*

*These experiments involve high intensity light sources and oxygen. It is the responsibility of each researcher to take appropriate safety precautions depending on their apparatus used to repeat this work. In particular, the LEDs should be housed in a suitable light-tight enclosure, and operators should wear goggles designed to work at the relevant wavelengths and to avoid skin exposure, particularly to red LEDs which have a higher penetration depth in human tissue. The work also involves the use of gaseous oxygen and organic solvents, and appropriate risk assessments should be carried out before conducting the reactions. In the work described here, the flow rate of O_2_ was measured and kept between 1 and 2.5 times stoichiometric. In the telescoped reactions, the open buffer tank between the two reactors allowed excess oxygen diffuse out of the MeOH solution. All experiments were performed in well-ventilated fume cupboards (minimum 80 FPM) in order to guarantee no accumulation of vapors, and in the case of the telescoped process, the photochemical and thermal reactors were located in separate vented cabinets (see the Supporting Information).*

## Data Availability

The data created by this research are available in the Supporting Information.

## Supporting Information Available

Experimental details, including emission of LEDs, photo-oxidation, and thermal condensation; NMR spectra; details of modeling (PDF)
