# Supplementary material for: Process Intensification of the Continuous Synthesis of Bio-Derived Monomers for Sustainable Coatings Using a Taylor Vortex Flow Reactor
Source: Org Process Res Dev. 2024 May 9;28(5):1917–28. doi: 10.1021/acs.oprd.3c00462 (PMC11110062; doi:10.1021/acs.oprd.3c00462)

Supporting Information

Process Intensification of the Continuous Synthesis of Bio-Derived Monomers for Sustainable Coatings using a Taylor Vortex Flow Reactor

Matthew D. Edwards,[a] Matthew T. Pratley,[a] Charles M. Gordon,[b] Rodolfo I. Teixeira,[a] Hamza Ali,[a] Irfhan Mahmood,[a] Reece Lester,[a] Ashley Love,[a] Johannes G. H. Hermens,[c] Thomas Freese,[c] Ben L. Feringa,[c] Martyn Poliakoff,[a] and Michael W. George[a]*

[a] *School of Chemistry, University of Nottingham, University Park, Nottingham NG7 2RD, UK*
[b] *Scale-up Systems Ltd., 23 Shelbourne Road, Dublin 4, D04 PY68, Ireland*
[c] *Advanced Research Centre CBBC, Stratingh Institute for Chemistry, Faculty of Science and Engineering, University of Groningen, Nijenborgh 4, 9747 AG Groningen, The Netherlands*

Table of Contents

1. Safety Note 1
2. Photo-oxidation of Furfural **1** in the PhotoVap 2
3. Photo-oxidation of Furfural **1** in the Taylor Vortex Reactor 2
4. General Reactor Operation – PhotoVortex – Small 3
5. Thermal condensation of **2** to desired alkoxybutenolide 4
6. Telescoped flow synthesis 6
7. Emission Spectra LEDs 7
8. Additional Optimization of PhotoVortex Photo-oxidation of 1 8
9. Infrared, Multivariate Curve Resolution (MCR), and Partial Least Squares Regression (PLS) Additional Information 8
10. Reaction Lab for Kinetic Modelling 9
11. NMR Characterization and Spectra 13

Safety Note: *These experiments involve high intensity light sources and oxygen. It is the responsibility of each researcher to take appropriate safety precautions depending on their apparatus used to repeat this work. In particular, the LEDs should be housed in a suitable light-tight enclosure and operators should wear goggles designed to work at the relevant wavelengths and to avoid skin exposure, particularly to red light LEDs which have a higher penetration depth in human tissue. The work also involves the use of gaseous oxygen and organic solvents and appropriate risk assessments should be carried out before conducting the reactions. In the work described here, the flow rate of O2 was measured and was kept between 1 to 2.5 times stoichiometric. In the telescoped reactions, the open buffer tank between the two reactors allowed excess oxygen diffuse out of the MeOH solution. All experiments were performed on well-ventilated fume cupboards (minimum 80 FPM) in order to guarantee no accumulation of vapors and, in the case of the telescoped process, the photochemical and thermal reactors were located in separate vented cabinets (See Below).*

Overview: Here we give further experimental details to complement those in the main paper. In particular, we show photographs of the reactors in operation, decribed the thermal reactor in some detail, provide emission data for the LEDs and show NMR data for the butenolide products.

Photo-oxidation of Furfural (1) in the PhotoVap

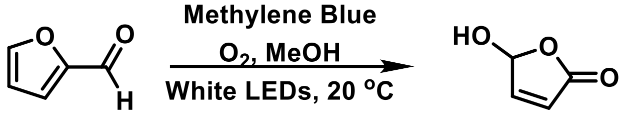

(1) (2)

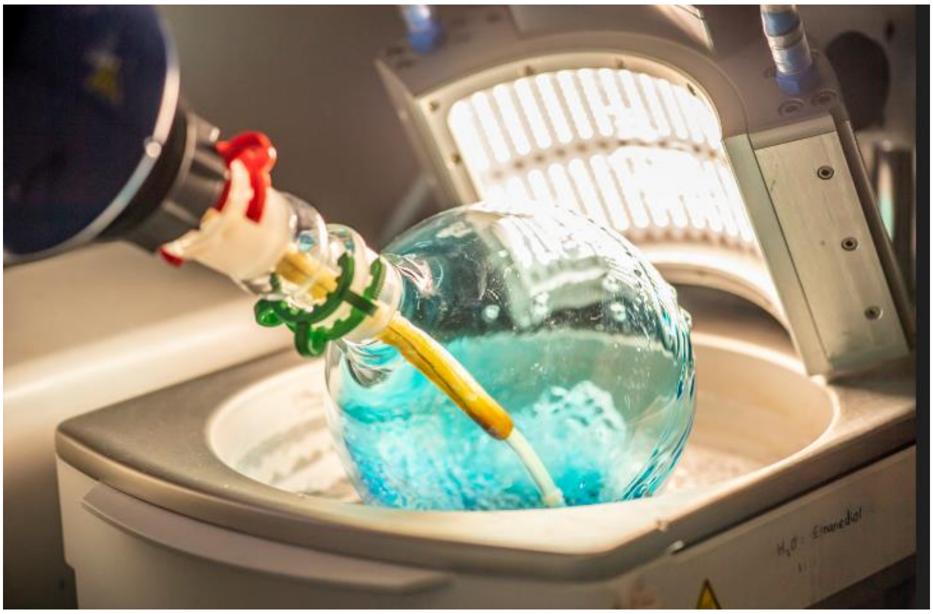

Fig. S1: General view of the PhotoVap with a 1 litre flask and a White LED assembly (Asynt Ltd) showing the tubes for filling and emptying the flask in the semi-continuous mode.

Photo-oxidation of Furfural (1) in the Taylor Vortex Reactor

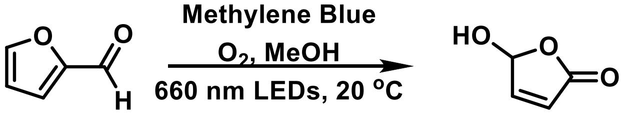

(1) (2)

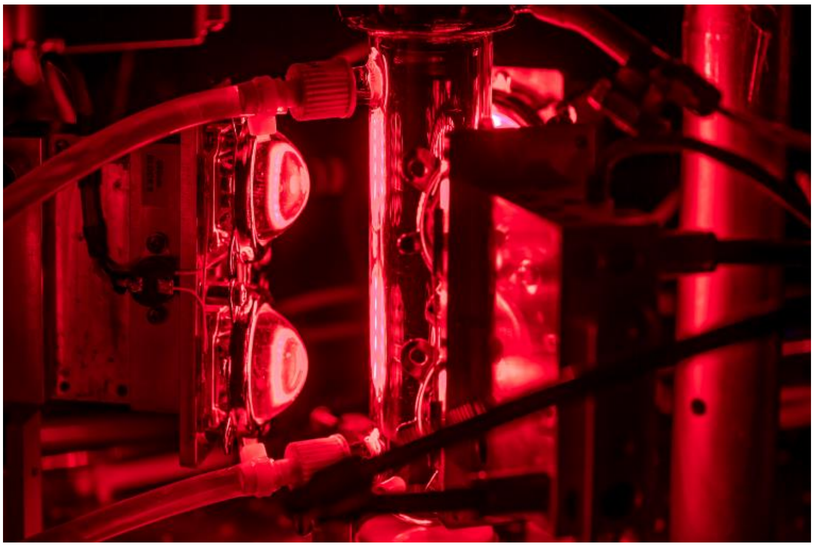

Fig. S2: Close-up view of the small vortex showing clearly one of the 200 W 660 nm LED assemblies with focusing lenses (in normal operation, the reactor uses three of these assemblies).

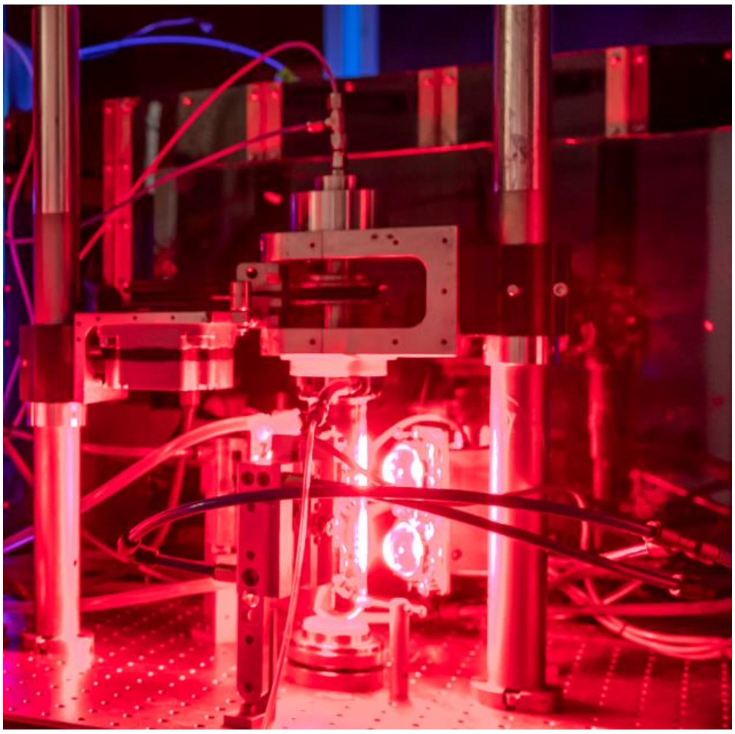

Fig. S3: A belt-driven version of the small PhotoVortex in operation with 660 nm LEDs inside a light-tight vented cabinet.

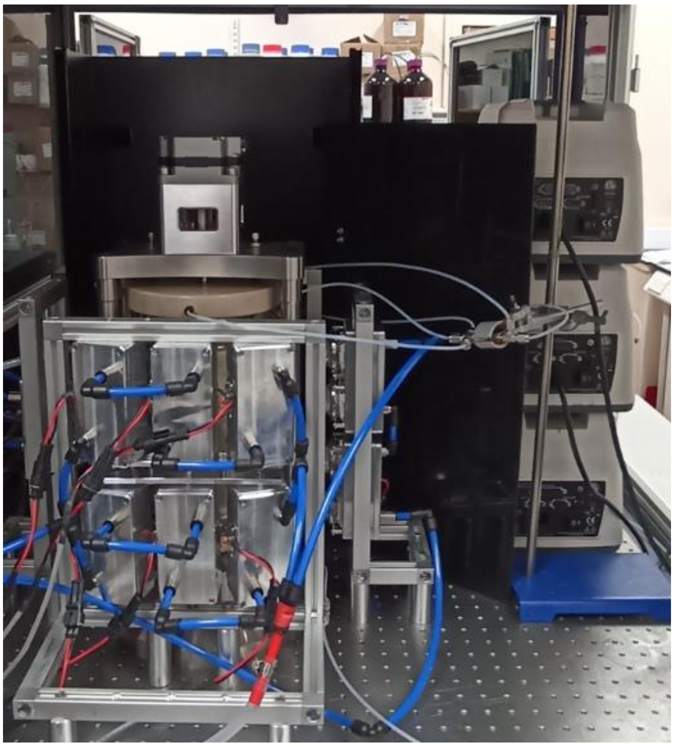

Fig. S4: The large PhotoVortex reactor surrounded by 15 × 200 W 660 nm LED assemblies (i.e. 3 kW in total). The peristaltic pumps can be seen on the right. The blue pipes supply coolant to the LEDs and the red cables supply the electrical power for the LEDs.

General Reactor Operation – PhotoVortex – Small

The recirculating chiller for the LEDs was turned on and set to 10 °C whilst the reactor chiller was set to the appropriate temperature for the reaction. The chillers were left to equilibrate for *ca.* 20 min. The reaction mixture was prepared as described below and the inlet tubing for the HPLC pump was fed into the solution

and the pump primed. The oxygen cylinder was opened and set to the desired pressure *ca.* 1 bar. The desired flowrate was set on the mass flow controller (MFC) and gas was pumped into the system at low flowrates (*ca.* 5 mL/min). The desired liquid flow rate was set on the HPLC pump and the solution was pumped into the reactor. The peristaltic pump to remove the products from the reactor was set to a flow rate suitably high to pump out the solution from the reactor. The rotation motor was turned on and slowly increased until the desired rotation speed was achieved. Then the LEDs were turned on at full brightness. The outlet peristaltic pump could then be fed into the ReactIR whereby steady state could be monitored. If no ReactIR was used, two full system volumes were allowed to pass before taking a sample for analysis; this ensured that the reactor had reached a steady state. Once the operation was complete, the LEDs were turned off and the reactor was flushed with solvent to clean at increased flowrates. The HPLC pump was turned off, and the rotation motor was slowly decreased to 0 rpm. The recirculating chillers were also turned off, providing the LED blocks were not excessively hot. The inlet pipe was switched to a container with a compatible solvent (usually the reaction solvent) and the reactor was flushed at 2-5 mL min^{-1}. The rotation speed was set to 200 rpm during this time and increased to 4000 rpm occasionally to ensure that all material was removed from the reactor. Once the reactor was clean, the motor, pumps, and MFCs were turned off and cylinder isolated.

Thermal condensation of (2) to desired alkoxybutenolide

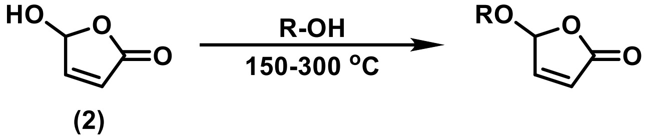

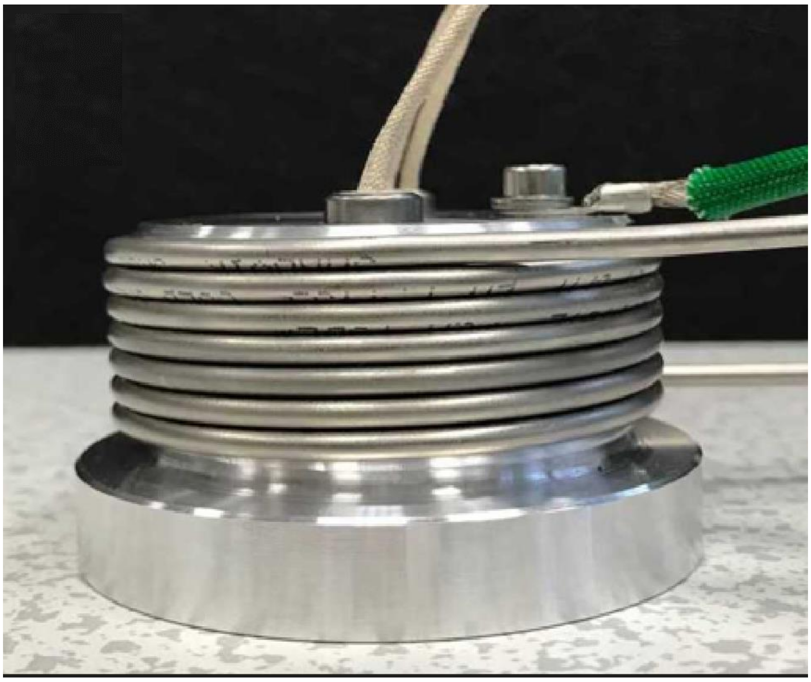

Fig. S5: Close up view of the 316 stainless steel thermal reactor (without its thermal insulation)

General Reactor Operation – Thermal tubular reactor

Maximum Operating Conditions:

The heated and pressurized parts of the reactor consist of 316 stainless steel (SS) tubing, provided by Swagelok (manufactured by Sandvik). The tubing is predominantly of 1/8" outer diameter (OD) and some sections are of 1/16" OD, with a wall thickness of 0.028" and 0.020", respectively. Swagelok quote maximum working pressures for these being 12,000 psig (~800 bar) for the 1/16" tubing and 8500 psig (~550 bar) for the 1/8" tubing. As such, the lower of these being 8500 psig must be taken as the maximum allowable working pressure of the system. Moreover, as the system is intended to be run with heat supplied to a coiled tube, temperature derating implications apply. Swagelok provide a derating factor for 316 SS tubing for up to 537 °C. The intended

use of the reactor is not likely to exceed 250 °C, at which, a derating factor of 0.79 is given for the closest value above this temperature (315 °C). As such, at this temperature, max. working pressure of 0.79 x 8500 psig = 6715 psig (~450 bar).

System Details:

The system has been designed for small-scale, continuous-flow organic synthesis in a variety of solvents, workable above their atmospheric pressure boiling points, under a pressurized system.

The system is comprised of a JASCO PU-980 pump, a JASCO BP-1580-81 backpressure regulator (BPR), a Swagelok sprung relief valve, and a reactor coil wrapped around an aluminum block, heated by two small cartridge heaters. The individual components are connected by a mixture of 316 SS tubing (OD, 1/16" – ¼"), fluorinated ethylene propylene (FEP) tubing (1/16"), with the relevant Swagelok, SSI, HIP, JASCO or Omni-Fit fittings connecting the components. Where required, the necessary pressure transducers and thermocouples are positioned and connected to a series of monitors/trips to ensure safe operation. In its current form, the Swagelok sprung relief valve is fitted with a spring designed to release pressure when the applied pressure is between 100-150 bar. Testing of the valve has shown the valve to release when the pressure reaches 150 bar.

In these superheated solvents, there is a significant change in density as the fluid is heated to high temperature so that the residence time cannot be calculated merely be dividing the reactor volume by the flow rate. On the other hand the flow rate of the cold solution entering the reactor can be measured quite accurately.

Pressure/Leak Test

As with the use of any high-pressure equipment, following any modification, maintenance or replacement of parts of the system, a pressure test should be conducted. This should be performed by pressurizing the system to at least 200 psi above the desired operating pressure of experiments, to ensure that there are no leaks before carrying out synthetic experiments.

Power Supply and Safety Trip:

The power supply for the cartridge heaters and JASCO pump should be connected to an extension lead which is plugged in to the trip box. This is to cause pumping of organic material to stop and heating of the reactor coil to stop if the pressure or temperature of the system goes too high. Via a trip loop, the trip box is also connected to a temperature monitor and pressure transducer indicator. The values of temperature and pressure at which these two monitors trip the system are set on the monitors interface. If these are exceeded, the trip will be activated, cutting the power to the pump and heaters. As an added precaution, the sprung relief valve in the system will release pressure if (in its current form) the system pressure reaches more than 150 bar. As such, the operating pressure of any reactions should be below this value and so the pressure trip should be somewhat lower. The maximum allowable pressures and temperatures are co-dependent and all operating conditions desired should be reviewed using the Swagelok Guides maximum pressure and the temperature derating at elevated temperatures. The safety trip also has a manual emergency stop button to allow the operator to cut the power to the connected components if necessary.

System Temperature:

The temperature of the heated reactor coil is controlled by two cartridge heaters placed within an aluminum block, around which the tubing is coiled (see Fig. S5). A K-type thermocouple is placed within a drilled cavity in the center of the block. This thermocouple is connected to a temperature monitor and control box. The temperature set using this box determines the heating supplied by the cartridge heaters until the set value is reached (and maintained). A second thermocouple is placed towards the outer edge of the heated block. This monitors the temperature at this position, giving a better indication of the temperature of the tubing where the reaction is occurring. In addition, this thermocouple is connected to the trip box in the event of the temperature of the reactor getting too high. Two other thermocouples are placed within the system, both are for monitoring purposes and are not connected to a trip. One is positioned at the outlet of the reactor to determine the temperature of the fluid as it exits the heated coil. The other is placed prior to the BPR inlet to ensure that the liquid has sufficiently cooled (to less than 60 °C) before entering the BPR.

System Pressure:

Pressure is introduced to the system with (in current form) a single JASCO PU-980 HPLC pump – though the system is designed for simple introduction of a second pump if required. A JASCO BPR is used to set the desired pressure for a reaction, and is automated to maintain the pressure at the set value (although blockage of the system could lead to higher pressures and tripping of the system). The pressure is monitored on both the pump and BPR. In addition, a pressure transducer is connected in the system (after the pump, prior to the BPR) to monitor the system pressure – this transducer is also connected to the trip box to cut power to the pump and heater in the event of over-pressure.

Choice of Solvents and Organic Materials:

Selection of reactor materials have been selected to appropriately have a wide compatibility range. 316 stainless steel shows good resistance to most substrates and tolerates wide pressure and temperature ranges without showing a decrease in performance detrimental to desired reaction conditions. Safety assessments prior to building the reactor concluded that 316 SS would be suitable at the temperatures and pressures desired for the reactions, and the nature of the halogenated materials to be used. However, due to the potential for corrosion, thorough cleaning of the reactor was performed after each use *i.e.* flushing through with *at least* two system volumes of appropriate solvent. Choice of seals where necessary (in the sprung relief valve) was made to maximize compatibility, leading to Kalrez seals being used which display high levels of compatibility with many chemical types.

Despite efforts to maximize the broad compatibility for reaction types, compatibility checks between materials with solvents/reagents should be conducted before reactions. Swagelok does not provide their own chemical-tubing compatibility data, although various other manufacturers provide data that can be used as a guide with an example provided at the end of this information (although comprehensive checks amongst data from various manufacturers should be consulted to reach a decision).

Telescoped flow synthesis

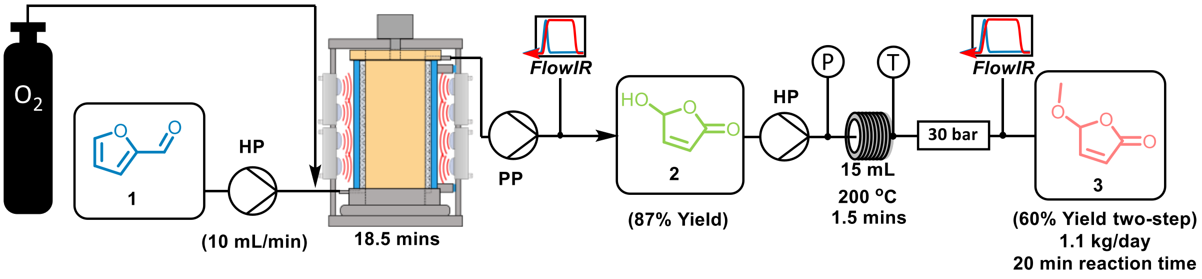

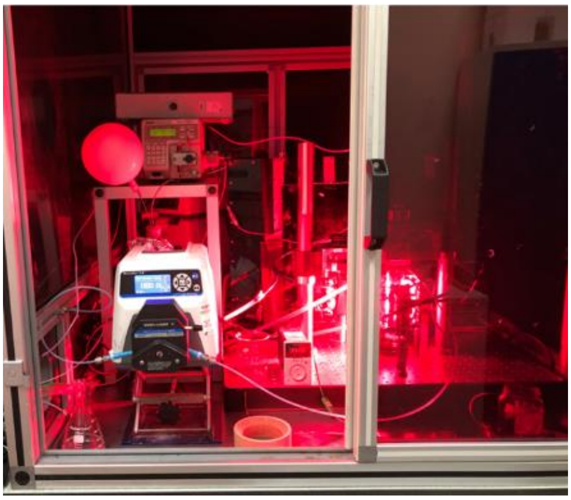

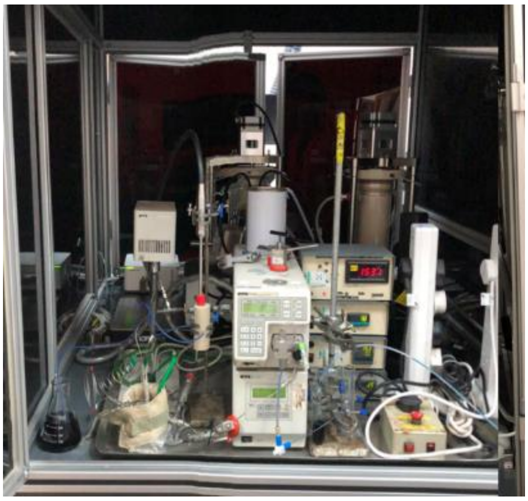

Fig. S6: Top – schematic of the larger scale telescoped photochemical and thermal reactions. Bottom – photographs of the actual equipment with the smaller vortex reactor. Note that the photochemical and thermal reactor assemblies and associated pumps are housed in completely separate vented cabinets with the reaction mixture pumped between them to ensure the safest possible operation in the presence of gaseous oxygen,.

Emission Spectra of LEDS: White LEDs (PhotoVap)

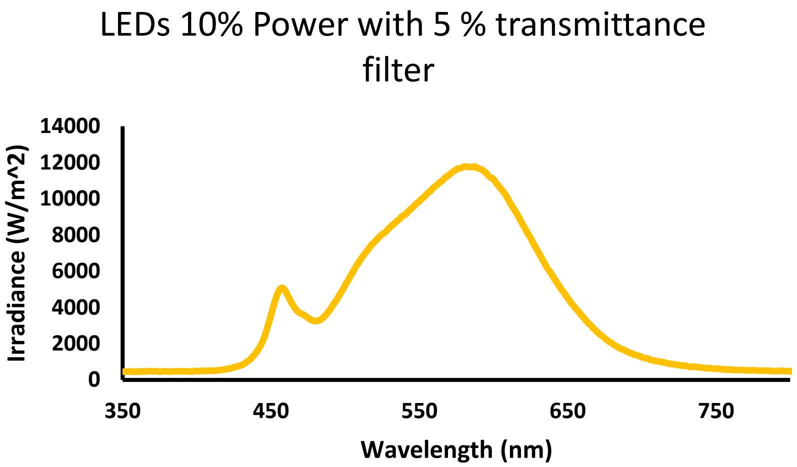

660 nm LED Blocks (Vortex Reactor) λ_{max} = 657 nm

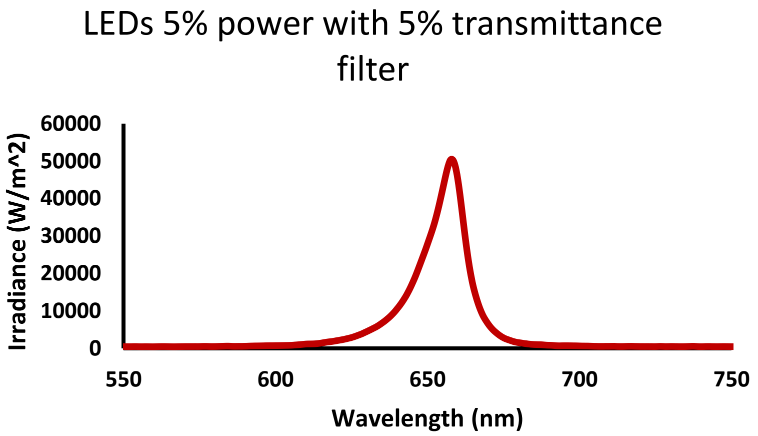

Emission of the White Light and 660 nm LEDs compared with the absorption bands of Methylene Blue; λ_{max} = 654 nm **(the intensities have been normalized)**

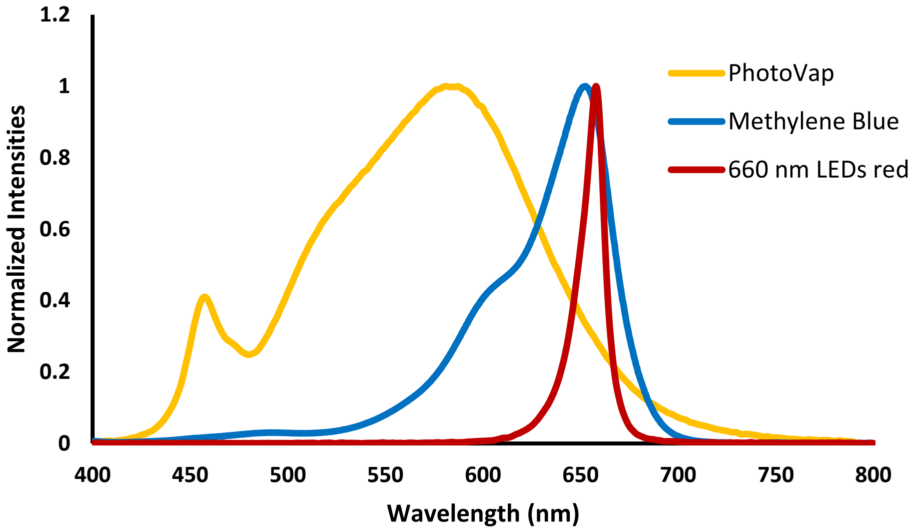

Additional Optimization of PhotoVortex Photo-oxidation of 1

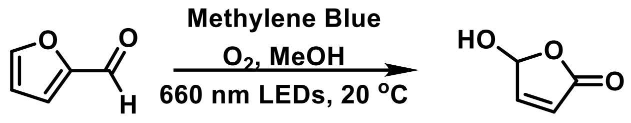

Entry	[Substrate] (M)	[Photocat] (M)	Spacetime (mins)	O_2 Eqiv.	Yield (%)
1	0.5	5	8	1.05	87
2	0.5	2	8	1.05	88
3	1	2	8	1.05	86
4	1	2	4	1.05	82
5	1	2	2.7	1.2	77
6	1	2	8	1.2	95
7	1	2	16	1.2	96

Yields are calculated *via* ^{1}H NMR with 1,3,5-trimethoxybenzene as internal standard. Space time is calculated for an irradiated volume of 8 mL. Rotor speed of 3000 rpm was chosen as optimal.

Additional Infrared, Multivariate Curve Resolution (MCR), and Partial Least Squares Regression (PLS) Information.

All infrared was collected with a custom-built flow cell using and a ReactIR 702L with a fibre optic probe. Pure reference spectra of the species in each reaction were collected at concentrations of 0.0, 0.5, 1.0, 1.5 and 2.0 mol L^{-1} to train the PLS and MCR models.

For both the MCR and PLS models, spectral pre-processing consisted of baseline correction, before normalising to the largest solvent peak. The solvent spectrum was then subtracted before trimming the spectrum to an appropriate region, (800 – 2000 cm^{-1}). These pre-processed spectra were then used to train the four component MCR and PLS models formed.

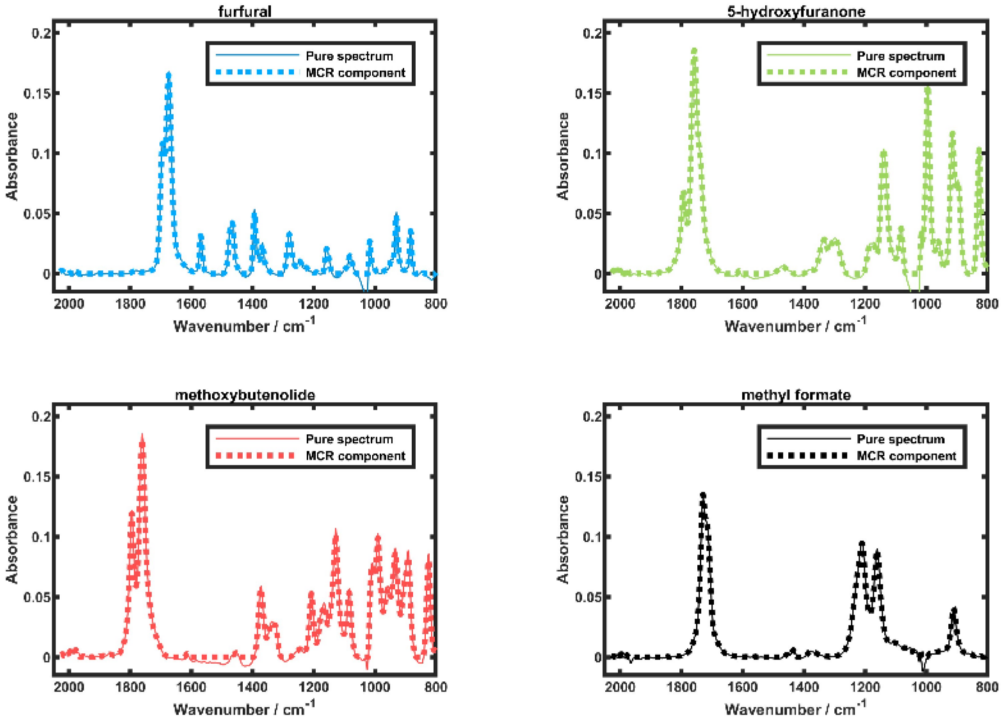

Fig. S7: Showing the pure spectra of furfural, 5-hydroxyfuranone, methoxybutenolide, and methyl formate in comparison to the MCR components.

Each reaction experiment consisted of taking a background air spectrum before a minimum of one solvent spectrum was collected by flowing the desired solvent through the system. The feed into the system was then swapped to a reaction mixture. Growth of the starting material was observed to plateau before the LED blocks were activated for the photochemical step, or the temperature increased for the thermal step. The spectra collected were processed as described above before being fed into the respective models enabling quantification of species.

Reaction Lab for Kinetic Modelling

The general workflow for generating a kinetic model in Reaction Lab is described below. The first step within the workflow is defining the various reaction steps that take place. In this case, the overall chemistry is defined in the scheme below.

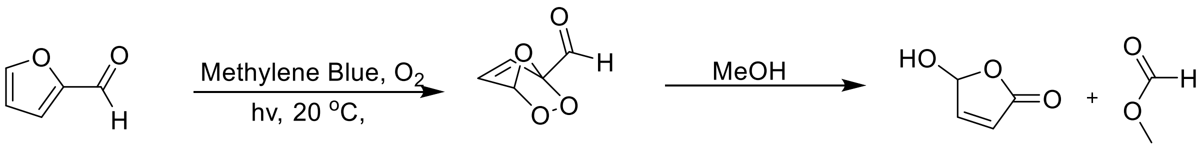

For the purposes of the Reaction Lab model, this was broken down into five individual reactions which were defined in the 'Reactions in Solution' section of the model as shown below, where FF is furfural and O_2 is triplet oxygen.

Reactions in Solution				
‖	Id	Reactants	→	Products
☐	rxn1	methylene_blue_1 + Photon	→	Methylene_blue_act
☐	rxn2	Methylene_blue_act + O2	→	Singlet_O2 + methylene_blue_1
☐	rxn3	FF + Singlet_O2	→	intermediate
☐	rxn4	intermediate + MeOH	→	5-hydroxyfuranone + MF
☐	rxn5	Singlet_O2	→	O2

Once the reaction scheme has been established, the 'Process Scheme' allows definition of an additional Headspace gas phase which allows simulation of the gas-liquid mass transfer of oxygen. We simulated the introduction of 'Photons' into the reactor by defining a 'photon feed tank' to the reactor which can be seen in the Figure below. The impact of light attenuation across the solution was not considered due to the very short pathlength for the light (~1 mm) in the reactor. Photon Flux was estimated as a function of power output from the LEDs. The total LED power was measured using calibrated Ocean Optics fiber optics and attenuating the LED output through a Neutral Density filter (0.1) Thor Labs® and a 1 mm aperture. The detector was placed at the outlet of the aperture and the light was collected. Utilizing the software in Ocean Optics operating in 'Absolute Irradiance' mode, an emission spectrum for the LED COB was obtained, integrated and back calculated to calculate the watts per LED chip. (*ca.* 45 W). This was multiplied by the number of chips mounted around the small-scale vortex reactor (6) providing a total photo-wattage of 270 W.

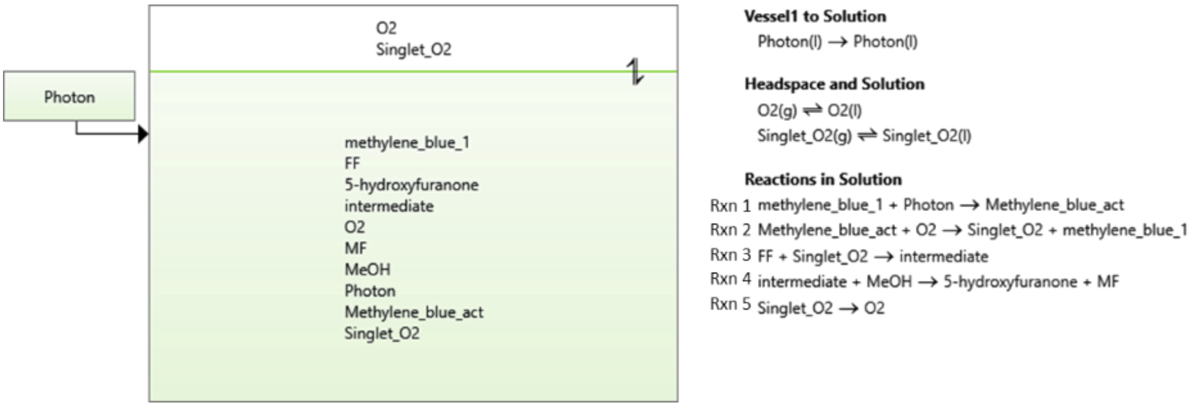

We needed to make some final assumptions and estimations before beginning our model development.

- We assumed a k_La value of 1 in the model, which corresponds to very fast gas → liquid mass transfer.
- The reaction volume was defined as 8.1 mL, which corresponds to the irradiated volume of the Taylor Vortex reactor.
- A Henry constant of 4.3 was obtained from the Dynochem database, based on O_2 solubility data in methanol at 20 ^oC.
- $t_{1/2}$ of $^1O_2$ in methanol ~1.4 x 10 $^{-5}$ s^{-1}
- Reactions 1 and 2 were defined as having very high rate constants (k = 1 x 10^8 L/mol/min)
- Reaction 3 was defined as being the rate determining step (RDS)
- The rate constant for Reaction 5 was determined from the literature value of $t_{1/2}$ for singlet O_2 in methanol

Reaction Lab has three key modules used in determining kinetic parameters and predicting the performance of the process:

<u>Fitting</u>

The Fitting module allows automated fitting of kinetic parameters to experimental data. This is achieved by varying the kinetic parameters from the reaction scheme, or other reaction parameters, with the aim of minimizing the difference between the predicted and experimental values. In the case of the photo-oxidation

reaction, only the rate constant was initially fitted. For later studies, k_La was also fitted using the Fitting module.

Simulator

The Simulator module allows simulation of the reaction based on defined kinetic parameters and reaction conditions. This allows manual adjustment of parameters such as k_La, photon flux, concentration, temperature, etc. and provides a visual estimate of what is happening in the reaction as input parameters are changed. For example, varying the k_La allows prediction of when the process will become mass transfer controlled (e.g. the rate of photo-oxidation is controlled by the rate of mass transfer of oxygen into the solution).

Optimization

The Optimization module allows the optimization of desired objective functions such as yield by varying any of defined input parameter ranges. This allows maximization, minimization, or targeting of a specific reaction outcome based on the current kinetic model and the user-defined parameter boundaries.

Workflow

Our initial analysis was based on a very limited data set based on the amounts of furfural, **1**, and hydroxyfuranone, **2**, present at two time points, determined by running the reaction at two different flow rates. A third data point for time = 0 was determined by the starting concentration of 2-furfural.

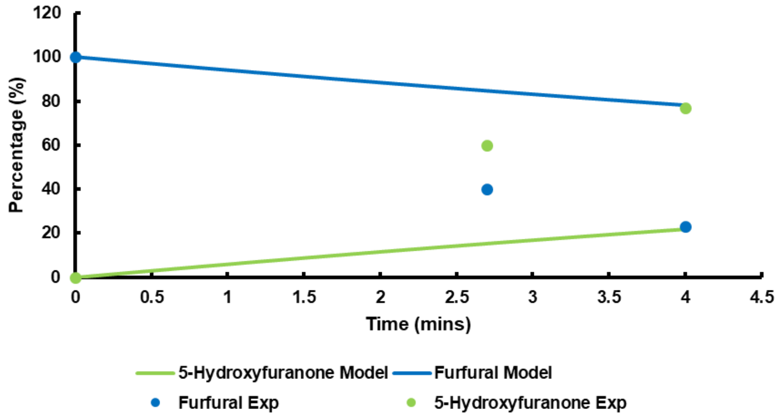

Phase/Flow	Reaction	Equation	Rate Exp.	kref	
Solution	rxn1	methylene_blue_1 + Photon → Methylene_blue_act	k [methylene_blue_1] [Photon]	k>	**1.0E+8** L/mol.min
Solution	rxn2	Methylene_blue_act + O2 → Singlet_O2 + methylene_blue_1	k [Methylene_blue_act] [O2]	k>	**1.0E+8** L/mol.min
Solution	rxn3	FF + Singlet_O2 → intermediate	k [FF] [Singlet_O2]	k>	**15000.0** L/mol.min
Solution	rxn4	intermediate + MeOH → 5-hydroxyfuranone + MF	k [intermediate] [MeOH]	k>	**100000.0** L/mol.min
Solution	rxn6	Singlet_O2 → O2	k [Singlet_O2]	k>	**2.97E+6** 1/min

We first used the Simulator module to make some initial guesses at the value of rate constant k> for rxn3. In this model, we assumed that as rxn3 is the rate-determining step it is the only one the value of which needs to be adjusted to fit the experimental data. Based on this, we then used the Fitting module to fit the value of the rate constants for rxn1 - rxn4 automatically. Rxn5 was omitted from the fitting, as this is a rate constant that has been previously confirmed in the literature values.

The outcome of the fitting is shown below. The visual fit to the experimental data was good, and the observation of a confidence interval of ±3.6% for Solution.rxn3.k> indicates that the predictivity for this rate constant is good. The 'Low Sensitivity' message for the other rate constants indicates that the quality of fit does not change significantly if the values of these parameters are varied, in agreement with our hypothesis.

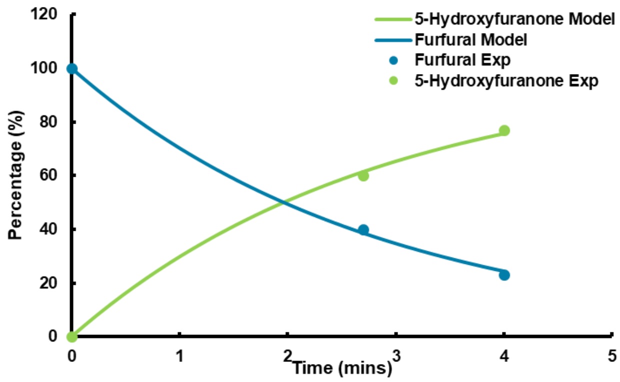

1	Solution.rxn1.k>	1.0E+8	Low Sensitivity	L/mol.min
2	Solution.rxn2.k>	1.0E+8	Low Sensitivity	L/mol.min
3	Solution.rxn3.k>	8.821E+4	+/- 3.6%	L/mol.min
4	Solution.rxn4.k>	1.0E+5	Low Sensitivity	L/mol.min

From here, we used the Optimization module to see whether we could validate the fitted reaction kinetics. We set a target yield value of 95% which we achieved as one of the data points in our experiment and varied the reaction end time (residence time for the flow experiment) using the Optimizer. Based on our initial two data points, Reaction Lab estimated that a reaction end time of 8.5 min. would be required to achieve 95% yield of **2** under the reaction conditions. This corresponds well to the actual value of 8.1 min. After collecting data for two further experiments, the model was updated and the optimization was repeated for a target of 95% yield of 5-hydroxyfuranone repeated. This time the model predicted an end time of 8.3 minutes, showing that with additional data points the quality of prediction had been improved slightly.

NMR Characterization and Spectra

NMR data for 5-hydroxy-2-(5H)-furanone (hydroxybutenolide) 2

Appearance: White solid

^{1}H NMR (400 MHz, $CDCl_3$) δ 7.33 (dd, *J* = 5.7, 1.2 Hz, 1H), 6.31 – 6.22 (m, 2H), 4.52 (s, 1H).

^{13}C NMR (101 MHz, $CDCl_3$) δ 171.87, 152.39, 124.65, 99.06.

NMR data for 5-methoxy-2-(5H)-furanone (methoxybutenolide) 3

Appearance: Colorless oil

^{1}H NMR (400 MHz, $CDCl_3$) δ 7.20 (dd, *J* = 5.7, 1.2 Hz, 1H), 6.22 (dd, *J* = 5.7, 1.2 Hz, 1H), 5.85 (t, *J* = 1.2 Hz, 1H), 3.55 (s, 3H).

^{13}C NMR (101 MHz, $CDCl_3$) δ 170.43, 150.34, 125.13, 104.15, 57.02.

NMR data for 5-ethoxy-2-(5H)-furanone (ethoxybutenolide) 4

Appearance: Colorless oil

^{1}H NMR (400 MHz, $CDCl_3$) δ 7.20 (dd, *J* = 5.7, 1.2 Hz, 1H), 6.21 (dd, *J* = 5.7, 1.2 Hz, 1H), 5.92 (t, *J* = 1.2 Hz, 1H), 3.92 (dq, *J* = 9.4, 7.1 Hz, 1H), 3.74 (dq, *J* = 9.4, 7.1 Hz, 1H), 1.26 (t, *J* = 7.1 Hz, 3H).

^{13}C NMR (101 MHz, $CDCl_3$) δ 170.63, 150.50, 125.00, 103.29, 66.22, 15.08.

NMR data for 5-isopropoxy-2-(5H)-furanone (isopropoxybutenolide) 5

Appearance: Colorless oil

^{1}H NMR (400 MHz, $CDCl_3$) δ 7.20 (dd, *J* = 5.7, 1.2 Hz, 1H), 6.22 (dd, *J* = 5.7, 1.2 Hz, 1H), 6.02 (t, *J* = 1.2 Hz, 1H), 4.11 (hept, *J* = 6.2 Hz, 1H), 1.29 (dd, *J* = 8.0, 6.2 Hz, 6H).

^{13}C NMR (101 MHz, $CDCl_3$) δ 170.84, 150.88, 124.76, 102.23, 73.77, 23.22, 22.04.

^{1}H NMR and ^{13}C NMR NMR Spectra of 5-hydroxy-2-(5H)-furanone (hydroxybutenolide) (2)

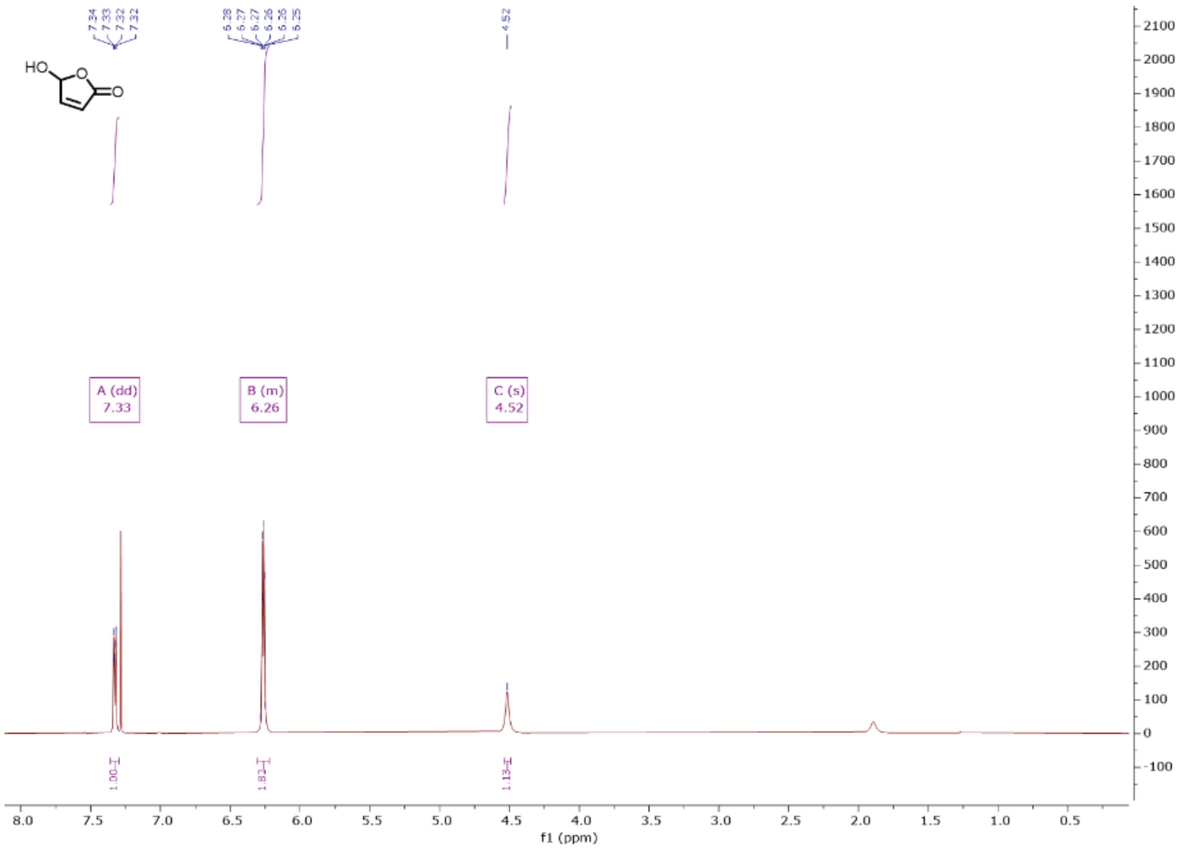

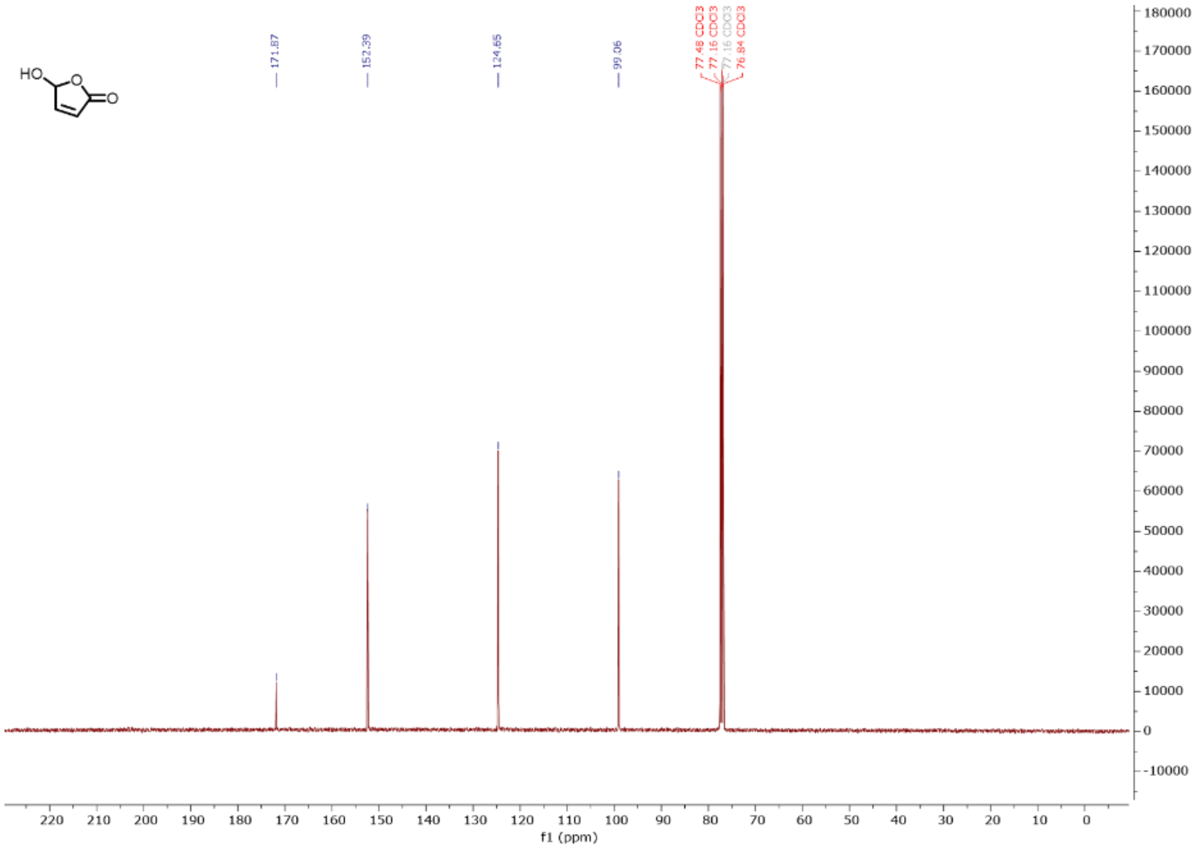

^{1}H NMR and ^{13}C NMR NMR Spectra of 5-methoxy-2-(5H)-furanone (methoxybutenolide) (3)

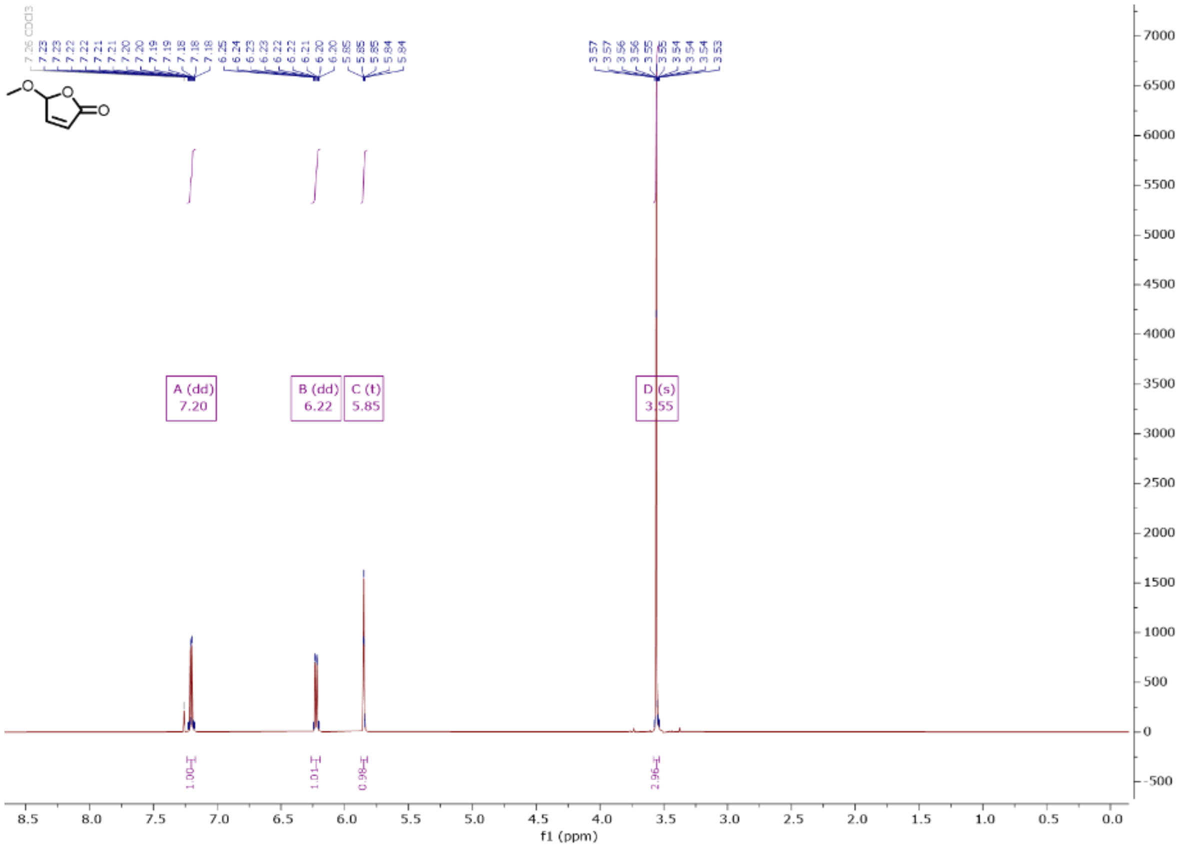

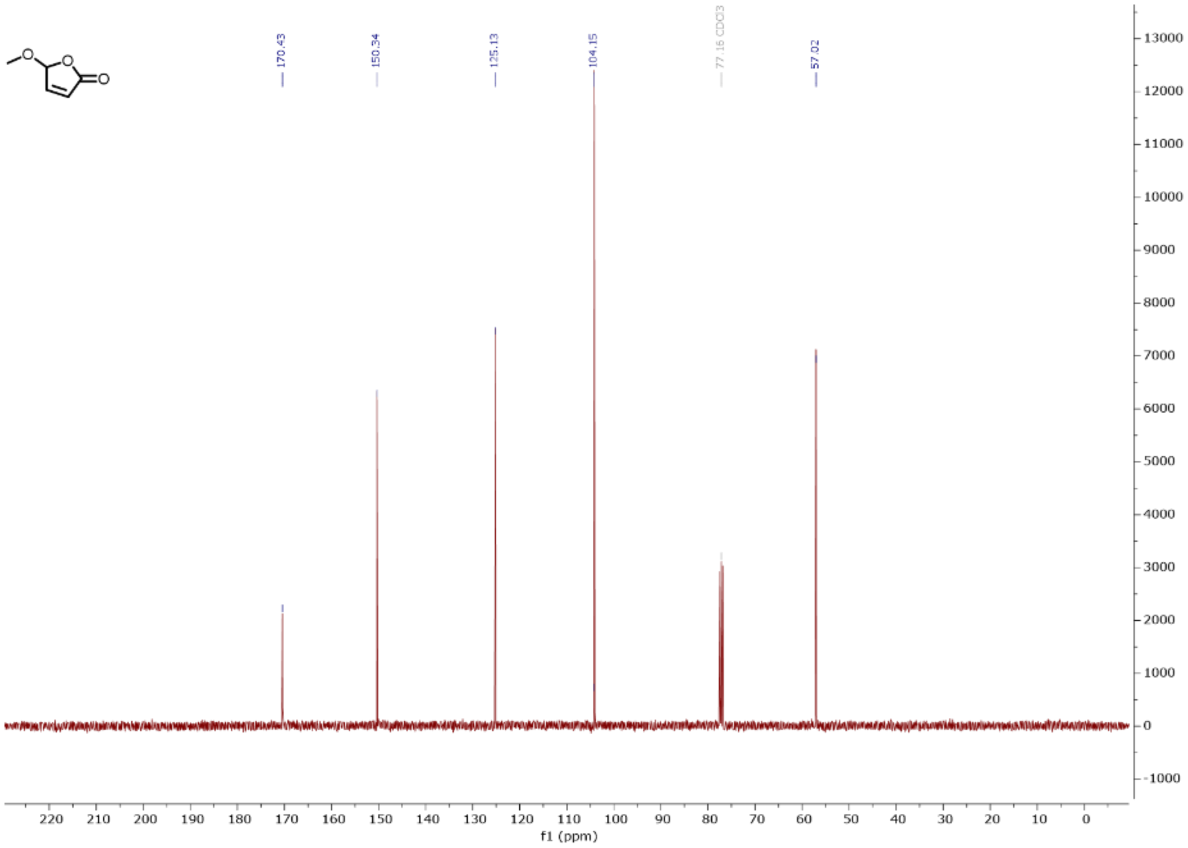

^{1}H NMR and ^{13}C NMR NMR Spectra of 5-ethoxy-2-(5H)-furanone (ethoxybutenolide) (4)

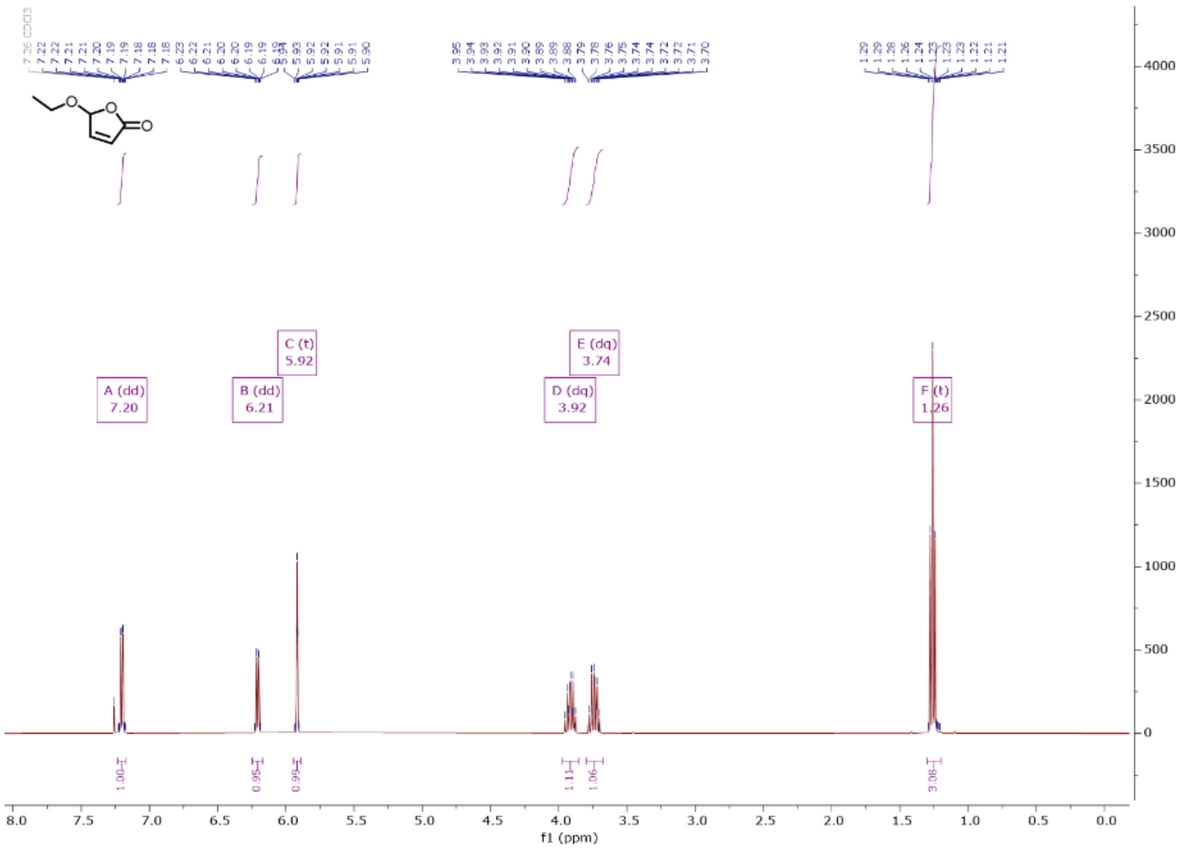

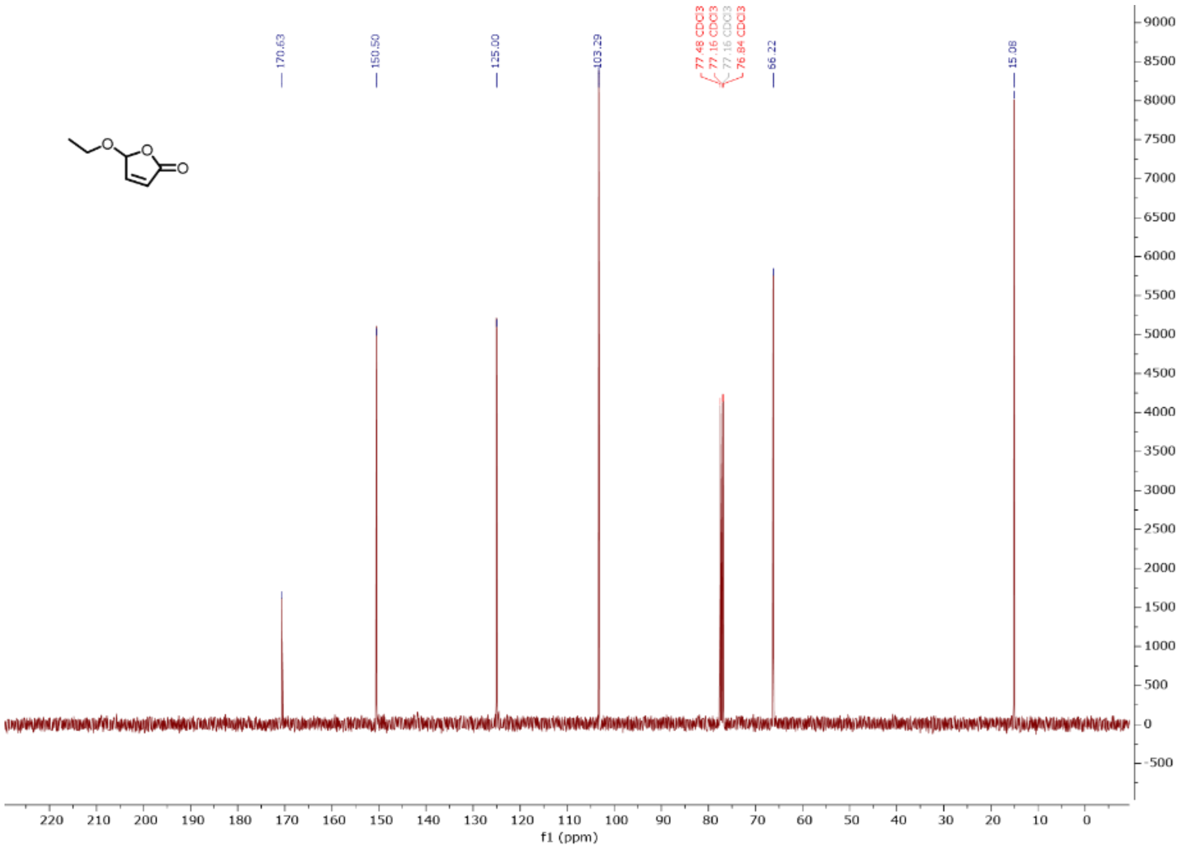

^{1}H NMR and ^{13}C NMR NMR Spectra of 5-isopropoxy-2-(5H)-furanone (isopropoxybutenolide) (5)

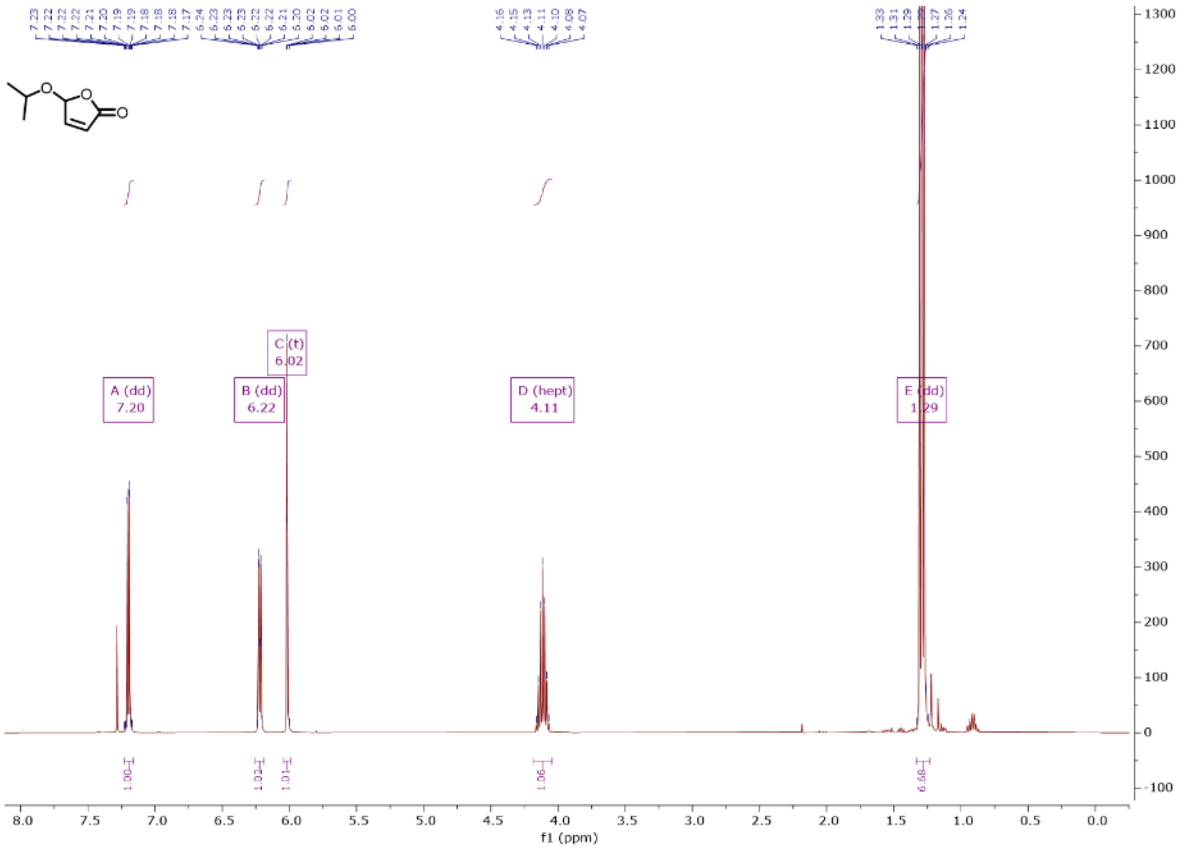

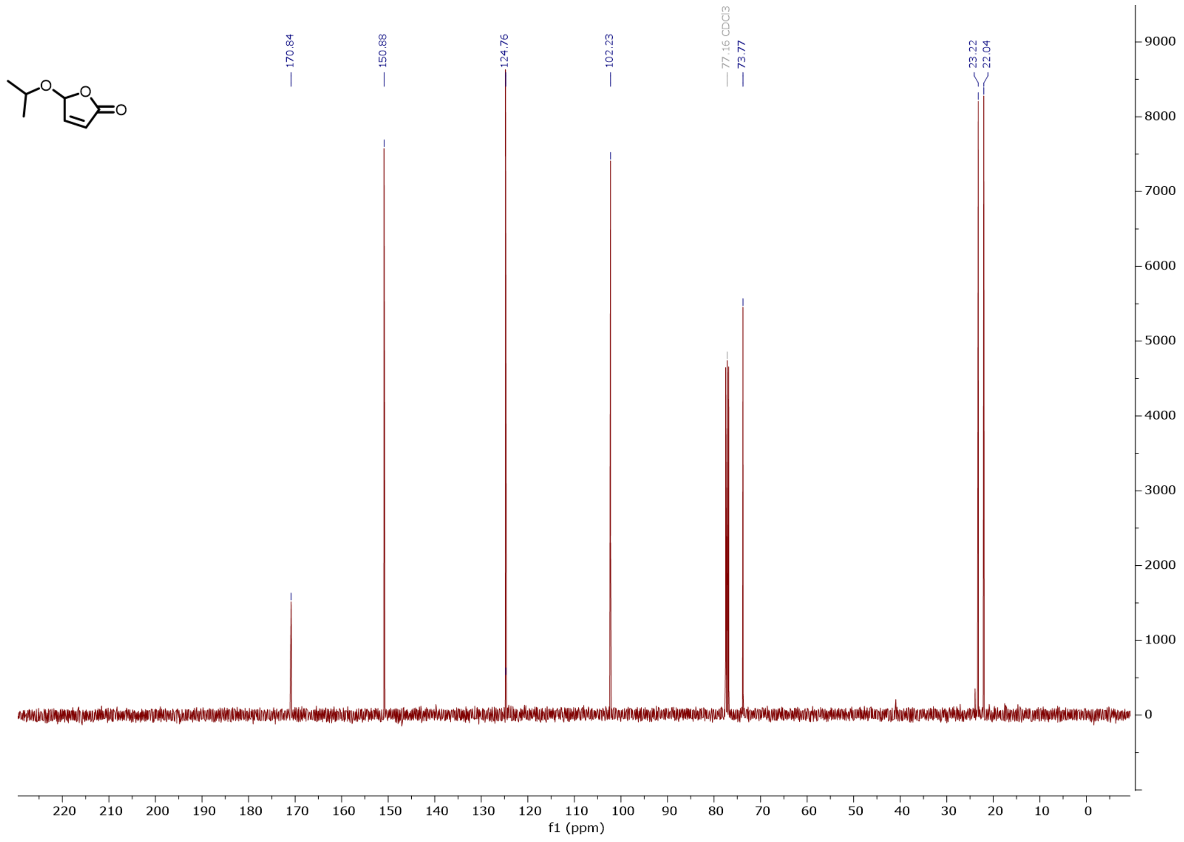

Supplement: Supplementary file 1 — op3c00462_si_001.pdf [file op3c00462_si_001.pdf]
